# VAPYRIN attenuates defence by repressing PR gene induction and localized lignin accumulation during arbuscular mycorrhizal symbiosis of *Petunia hybrida*

**DOI:** 10.1111/nph.17109

**Published:** 2020-12-25

**Authors:** Min Chen, Sébastien Bruisson, Laure Bapaume, Geoffrey Darbon, Gaëtan Glauser, Martine Schorderet, Didier Reinhardt

**Affiliations:** ^1^ Department of Biology University of Fribourg Fribourg CH‐1700 Switzerland; ^2^ Neuchâtel Platform of Analytical Chemistry University of Neuchâtel Neuchâtel 2000 Switzerland

**Keywords:** arbuscular mycorrhiza, defence, lignin, pathogenesis‐related protein, *Petunia hybrida*, *Rhizophagus irregularis*, symbiosis, VAPYRIN

## Abstract

- The intimate association of host and fungus in arbuscular mycorrhizal (AM) symbiosis can potentially trigger induction of host defence mechanisms against the fungus, implying that successful symbiosis requires suppression of defence.
- We addressed this phenomenon by using AM‐defective *vapyrin* (*vpy*) mutants in *Petunia hybrida*, including a new allele (*vpy‐3*) with a transposon insertion close to the ATG start codon. We explore whether abortion of fungal infection in *vpy* mutants is associated with the induction of defence markers, such as cell wall alterations, accumulation of reactive oxygen species (ROS), defence hormones and induction of pathogenesis‐related (PR) genes.
- We show that *vpy* mutants exhibit a strong resistance against intracellular colonization, which is associated with the generation of cell wall appositions (papillae) with lignin impregnation at fungal entry sites, while no accumulation of defence hormones, ROS or callose was observed. Systematic analysis of PR gene expression revealed that several PR genes are induced in mycorrhizal roots of the wild‐type, and even more in *vpy* plants. Some PR genes are induced exclusively in *vpy* mutants.
- Our results suggest that *VPY* is involved in avoiding or suppressing the induction of a cellular defence syndrome that involves localized lignin deposition and PR gene induction.

## Introduction

Arbuscular mycorrhiza (AM) is a mutualistic association of the majority of land plants with fungi of the subphylum *Glomeromycotina* (Smith & Read, 2008; Spatafora *et al*., 2016), which confers various benefits to the plant host (Chen, M *et al*., 2018). Although AM require mutual recognition of the partners to establish intracellular compatibility, the interaction is characterized by a very low degree of host specificity (Smith & Read, 2008). For example, the AM fungal model species *Rhizophagus irregularis* can colonize all angiosperms that were tested with few exceptions, represented by plant taxa that are generally incapable of engaging in AM (e.g. the Brassicaceae, including oilseed rape and *Arabidopsis thaliana*).

A striking aspect of the strong compatibility in AM is the massive intracellular colonization of the root cortex by hyphae, arbuscules and vesicles, which can result in > 90% colonization of the entire root system of the host. AM fungi share typical fungal cell wall components (e.g. chitin) with fungal pathogens, and plants have very sensitive detection mechanisms for such general microbial molecules, which are known as pathogen‐associated molecular patterns (PAMPs) or, more generally, as microbe‐associated molecular patterns (MAMPs) (Boller & Felix, 2009). Hence, the abundance of fungal material in the root, and the intimate interaction between the two symbiotic partners imply that disease resistance mechanisms in the host must be under tight control to avoid defence reactions to be triggered against AM fungi (Gianinazzi‐Pearson, 1996; Gianinazzi‐Pearson *et al*., 1996; Marsh & Schultze, 2001; García‐Garrido & Ocampo, 2002; Zipfel & Oldroyd, 2017).

Several studies have reported induction of defence mechanisms at early stages of AM interactions (Gianinazzi‐Pearson *et al*., 1996; Kapulnik *et al*., 1996; Campos‐Soriano *et al*., 2010; Marcel *et al*., 2010). After initial induction, these defence responses usually become repressed to, or below, the responses in noninoculated control roots. Interestingly, in simultaneous inoculations, AM fungi can reduce the induction of defence responses elicited by a pathogen (Guenoune *et al*., 2001) or by a chemical inducer of defence (David *et al*., 1998). This indicates that infection by AM fungi is associated with active suppression of defence. Indeed, an AM fungal effector protein that promotes biotrophic compatibility in host plants by suppressing defence has been identified in *R*. *irregularis* (Kloppholz *et al*., 2011). On the other hand, it was shown in many cases that AM fungi mediate increased disease resistance in roots as well as in the aerial parts of colonized plants, a phenomenon known as mycorrhiza‐induced resistance (MIR), involving a mechanism that resembles induced systemic resistance (ISR) (Jung *et al*., 2012; Pieterse *et al*., 2014).

Characteristic markers of defence include the stress hormones salicylic acid (SA; Loake & Grant, 2007), jasmonic acid (JA; Browse, 2009; Wasternack & Hause, 2013) and ethylene (van Loon *et al*., 2006a), cell wall reinforcements such as callose and lignin (Millet *et al*., 2010; Miedes *et al*., 2014; Chowdhury *et al*., 2016; Liu *et al*., 2018), induction of reactive oxygen species (ROS) (Jones & Dangl, 2006), and the induction of pathogenesis‐related (PR) proteins that are thought to contribute to disease resistance (van Loon *et al*., 2006b). Indeed, several of these defence markers are induced in mycorrhizal roots, and the observation that AM fungi express ROS scavenging enzymes during symbiosis (Lanfranco *et al*., 2005) is compatible with the view that they face defence mechanisms, in particular in mutants with compromised symbiosis competencies (Gianinazzi‐Pearson *et al*., 1996; Marsh & Schultze, 2001). However, in this context it is important to note that most of our knowledge on plant defence mechanisms was gained in the shoot (mainly from leaves), while defence mechanisms in the roots are considerably different (Chuberre *et al*., 2018), and have been explored to a lesser extent.

We have previously described two allelic mutants in petunia (*Petunia hybrida*), *penetration and arbuscule morphogenesis1‐1* (*pam1‐1*) and *pam1‐2*, which carry transposon insertions in the *VAPYRIN* (*VPY*) gene (hereinafter referred to as *vpy‐1* and *vpy‐2*, respectively). Detailed phenotypic analysis showed that *vpy* mutants are defective in infection of hypodermal cells and in arbuscule formation, indicating that VPY is required for intracellular accommodation of AM fungi during symbiosis. VPY function is conserved between petunia and *Medicago truncatula* (Pumplin *et al*., 2010), and acts downstream of calcium spiking, the central element in symbiotic signalling (Murray *et al*., 2011). Interestingly, VPY protein is localized to small mobile compartments (*VPY* bodies), which are thought to be involved in cellular trafficking during symbiosis (Feddermann *et al*., 2010; Pumplin *et al*., 2010; Zhang *et al*., 2015; Bapaume *et al*., 2019; Liu *et al*., 2019), and during protonema development in the moss *Physcomitrella patens* (Rathgeb *et al*., 2020). The fact that the AM fungus in *vpy* mutants exhibits conspicuous deformations upon cell penetration, and forms hyphal septa (a sign of stress) (Sekhara Reddy *et al*., 2007; Feddermann *et al*., 2010), suggests that this trafficking pathway may be involved, directly or indirectly, in modulating defence during intracellular stages of AM.

Here, we describe a new mutant allele, *vpy‐3*, which is a null allele, as a result of a *dTph1* transposon insertion after only eight codons from the start codon. *Vpy‐3* exhibits similar defects as the two other *vpy* alleles, indicating that these also represent functional null alleles. Microscopic and molecular analysis using a range of defence markers indicates that the abortion of the AM fungus in *vpy* mutants involves a cellular defence response that is independent of callose deposition and of the classical stress hormones SA, JA and ethylene, but is associated with induction of several PR genes, and with cell wall lignification during intracellular invasion.

## Materials and Methods

### Plant lines, fungal material and growth conditions

Seeds of the petunia transposon line W138 or wild‐type W115 were germinated on seedling substrate (Klasmann‐Deilmann Europe GmbH, Geeste, Germany; http://www.klasmann‐deilmann.com). For the mutant screen, plantlets were transferred to a sterilized mixture of 75% sand with 25% unfertilized soil (also referred to as sand substrate), inoculated with *c*. 10 g of pot culture inoculum of *Rhizophagus irregularis* (MUCL 43204), and cultured as previously described (Nouri *et al*., 2014). For all experiments involving mutants, the plants were inoculated with nurse plants by co‐culturing in the same pot petunia plants with chive plants (*Allium schoenoprasum*) that had been inoculated at least 4 wk before with *R*. *irregularis*. Plants were grown in growth chambers under 12 h : 12 h, 25°C : 20°C, light : dark conditions.

### Isolation of the *vpy‐3* mutant allele

The *vpy‐3* allele was isolated as previously described (Sekhara Reddy *et al*., 2007; Rich *et al*., 2015). Briefly, eight individuals per segregating family were assessed for mycorrhizal colonization after 5 wk of colonization with *R*.* irregularis* (primary screen). Root samples were taken from inoculated plants, stained with trypan blue and screened visually for the presence of AM fungal structures. Families with AM‐defective individuals were further grown for seed production, and additional seeds of the respective family were sown for phenotypic analysis and assessment of the segregation pattern (secondary screen). Homozygous mutant individuals of the new mutant line were crossed with *pam1‐1* (*vpy‐1*) and *pam1‐2* (*vpy‐2*) (Feddermann *et al*., 2010). One hundred per cent of the F_1_ progeny showed the mutant phenotype of the parents, indicating that *vpy‐3* is allelic to the previous *vpy* mutants. Isolation of the *vpy‐3* locus by PCR, cloning into pGEMT, and Sanger sequencing revealed an insertion of a *dTph1* copy after 25 nucleotides from the start codon (ATG). For detailed phenotypic analysis, the *vpy‐3* allele was stabilized by segregating out the active translocator locus *ACT1* after crossing with the stabilizer line W5 (Stuurman & Kuhlemeier, 2005).

### Assessing AM fungal colonization and papilla formation

For primary mutant screening, roots were harvested, washed and stained with trypan blue (0.01% w/v) in 0.5% (v/v) acetic acid for 10 min at 95°C, and washed with water for visual inspection. For secondary screening, roots were cleared in 10% KOH (30 min at 95°C), washed twice with water, stained for 10 min with trypan blue staining solution at 95°C (20% glycerol, 30% lactic acid and 0.01% Trypan blue) and rinsed twice with 10% lactic acid.

For initial assessment of fungal hyphae and cell wall papillae in *vpy‐3*, plants were inoculated with *R*. *irregularis* in nurse plant chambers for 4 wk. Roots were fixed for 2 h at room temperature in 4% (v/v) paraformaldehyde. After several washes, roots were stained overnight at 4°C in the dark with 5 µg ml^−1^ wheat germ agglutinin (WGA) coupled to fluorescein isothiocyanate (ThermoFisher Life Technologies, Carlsbad, CA, USA) in Soerensen's phosphate buffer (0.133 M, pH = 7.2). Before mounting, samples were incubated for 10 min in 50 µg ml^−1^ propidium iodide at room temperature. In a second experiment for quantification of papilla formation in all *vpy* alleles, plants were inoculated with *R*.* irregularis* in nurse plant chambers for 4 wk, then roots were harvested and cleared in 10% KOH for 20 min. After four washes with deionized water, roots were stained overnight with WGA‐Alexa488 (ThermoFisher Life Technologies) in Soerensen's phosphate buffer (0.133 M, pH = 7.2), followed by counterstaining with 0.2% basic fuchsin (857343; Sigma). For microscopy, the roots were immersed in a modified version of ClearSee (Kurihara *et al*., 2015) containing 10% (w/v) xylitol, 25% (w/v) urea and 2% (w/v) sodium dodcyl sulphate. Images were acquired on a Leica SP5 confocal microscope.

### Callose and lignin staining

For callose detection, roots inoculated by nurse plants were fixed overnight with 4% (v/v) paraformaldehyde (Supporting Information Fig. S6, see later), or with 1 : 3 acetic acid : ethanol (Table S1), washed five times with water, followed by staining with 0.01% (w/v) aniline blue in 150 mM KH_2_PO_4_ (pH = 9.5) for 48 h. Lignin was stained with phloroglucinol solution (100 ml 95% EtOH, 16 ml concentrated HCl, 0.1 g phloroglucinol) for 30 min and analysed directly. Callose epifluorescence images and lignin accumulation were analysed with a Leica DMR microscope (Leica Microsystems, Heerbruck, Switzerland), equipped with an Axiocam (Zeiss, Oberkochen, Germany).

### Detection of ROS

For detection of hydrogen peroxide (H_2_O_2_), roots inoculated from nurse plants were harvested and treated with a freshly prepared solution of 1 mg ml^−1^ 3,3′‐diaminobenzidine (DAB) in 10 mM MES‐NaOH (pH 5.6) (Salzer *et al*., 1999). Preparing the solution requires intense stirring and careful acidification with HCl to around pH = 3 (to increase DAB solubility) followed by buffering with MES‐NaOH. After overnight incubation at room temperature in the dark, roots were washed four times with water and incubated in ClearSeeD for microscopic analysis. For detection of O_2_^–^, roots were treated for 1 h at room temperature in the dark with a fresh solution of 1mg ml^−1^ nitroblue tetrazolium (NBT) in 10 mM phosphate buffer, pH = 7.8. After four washes with water, roots were incubated in ClearSeeD for microscopic analysis.

### Electron microscopy and preparation of semithin sections

Roots inoculated from nurse plants were fixed for 2 h at room temperature in 4% (v/v) glutaraldehyde and postfixed with 1% (w/v) OsO_4_ at 4°C overnight. Further processing of the samples and embedding in Spurr's resin was carried out as previously described (Spurr, 1969). For immunocytochemical analysis, tissues were embedded in Lowicryl K4 (Sigma‐Aldrich) as described (Altman *et al*., 1984) (Methods S1). Semithin sections (1 µm) were stained with 1 % (w/v) toluidine blue in 1% (w/v) borax (Na_2_B_4_O_7_). Images were acquired in the bright field mode on a Leica DMR microscope equipped with an Axiocam (Zeiss). For transmission electron microscopy (TEM) analysis, ultrathin sections (70 nm) were prepared on a Reichert‐Jung Ultracut E (Leica Microsystems). Contrasting was performed with 2% (w/v) uranyl acetate (UO_2_(CH_3_COO)_2_) and lead citrate solution prepared according to Reynolds (1963). Images were acquired on a Philips Biotwin CM100 (FEI Inc., Hillsboro, OR, USA).

### Identification of lignin biosynthetic genes and PR genes

Lignin biosynthetic genes with a potential role in roots (Vanholme *et al*., 2010) were identified from a list of genes annotated as lignin‐related in a previous expressed sequence tag sequencing project (Breuillin *et al*., 2010). The full‐length gene sequences were then identified from the predicted transcriptome of *P*.* axillaris* at the SolGenomics database (https://solgenomics.net). PR gene homologues were identified by searching the *Petunia axillaris* predicted transcriptome at the SolGenomics database (https://solgenomics.net) using tobacco PR proteins (van Loon *et al*., 2006b) by tblastn. Primers for quantitative real‐time reverse‐transcriptase polymerase chain reaction (qRT‐PCR) were designed by the Primer3 tool (https://bioinfo.ut.ee/primer3‐0.4.0/) (see Tables S2, S7). Preliminary analysis of gene expression was performed in mycorrhizal and nonmycorrhizal roots of wild‐type and *vpy* mutant roots to identify genes that were expressed in any of the tested conditions, and these were used for further qRT‐PCR analysis.

### Determination of SA and JA

Concentrations of JA and JA‐isoleucine (JA‐Ile) were determined by ultrahigh performance liquid chromatography‐tandem MS (UHPLC‐MS/MS) according to Glauser *et al*. (2014). SA and conjugated SA were separated by two‐phase extraction, followed by acid hydrolysis of conjugated SA, and quantification as previously described (Fragniere *et al*., 2011).

### Supplementary materials

*M. truncatula* growth conditions and experimental procedures (Broughton & Dillworth, 1971), as well as immunostaining procedures, manipulation of RNA, qRT‐PCR (Pfaffl, 2001) and statistical analyses, are described in Notes S1 and Methods S1.

## Results

### Isolation of a new *vpy* allele

**Fig. 1:**
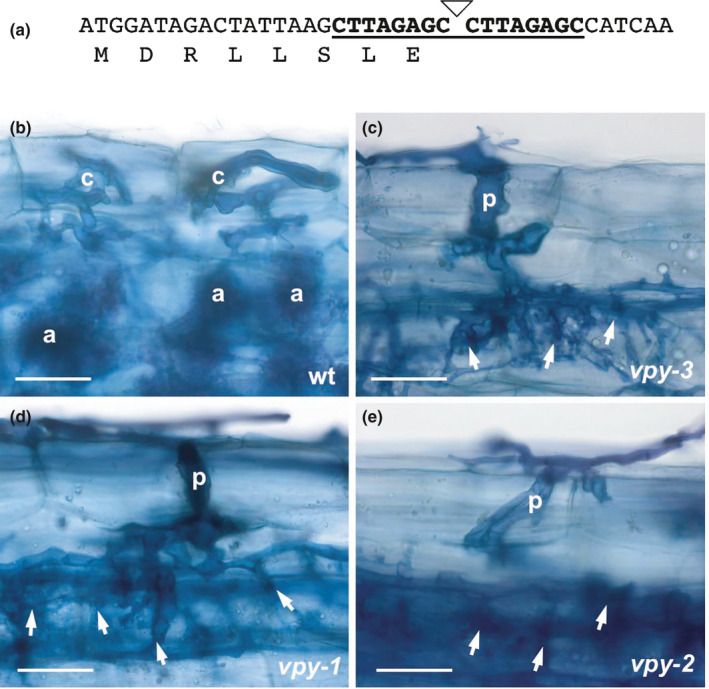
Arbuscular mycorrhizal (AM)‐defective phenotype and molecular characterization of *vpy‐3* in *Petunia hybrida*. (a) Molecular aspects of the new *vpy‐3* allele. The petunia *VAPYRIN* coding sequence is shown from the ATG start codon to the insertion site of a *dTPh1* transposon (triangle). The 8 bp target site duplication is in bold and underlined. Because of stop codons in all three reading frames of the *dTPh1* sequence, the truncated protein can be predicted to contain only eight residual amino acids of VAPYRIN. (b) The colonization pattern in the wild‐type (wt) revealed by trypan blue staining. Infection by hyphal coils in hypodermal cells, and arbuscules in the cortex. (c–e) Colonization pattern in *vpy* mutants revealed by trypan blue staining. Infection by enlarged penetration pegs, and subsequent profusely branched hyphal colonization (arrows) without arbuscules in *vpy‐3* (c), and for comparison, *vpy‐1* (d) and *vpy‐2* (e). a, arbuscules; c, hyphal coils; p, penetration peg; arrows indicate aberrant hyphal colonization in the cortex. Bars, 50 µm.

In a forward genetic screen for AM‐defective mutants, we isolated a mutant candidate with severely decreased colonization of the AM fungus *Rhizophagus irregularis*. Colonization of the root surface resulted in the production of deformed hyphopodia, and penetration of the epidermis and hypodermis was often arrested. Based on the phenotypic similarity with the previously isolated mutants *pam1‐1* and *pam1‐2* (Sekhara Reddy *et al*., 2007; Feddermann *et al*., 2010), we crossed the new mutant with these *pam1* alleles to test for allelism. One hundred per cent of the F_1_ progeny showed the same AM‐resistant phenotype as the two parents (data not shown), confirming that the new mutant is allelic to *pam1‐1* and *pam1‐2*. As the encoded protein has since been named VAPYRIN (VPY) as a result of its domain structure (Feddermann *et al*., 2010; Pumplin *et al*., 2010; Feddermann & Reinhardt, 2011; Murray *et al*., 2011), we further refer to the new allele as *vapyrin‐3* (*vpy‐3*), and the previously isolated alleles as *vpy‐1* and *vpy‐2*, respectively. The coding region of the *VPY* gene in the *vpy‐3* mutant was amplified by PCR and cloned in order to identify the nature of the mutation. Indeed, a *dTph1* insertion was detected at position 25 from the predicted start codon (ATG), leaving only eight of the 535 amino acids of the predicted protein (Feddermann *et al*., 2010) (Fig. 1a), suggesting that *vpy‐3* represents a null allele.

Initial phenotypic analysis showed that all three *vpy* mutants showed very low amounts of overall root colonization (< 10%) after inoculation with pot inoculum (spores and fragmented mycorrhizal root), as shown before for *vpy‐1* and *vpy‐2* (Sekhara Reddy *et al*., 2007). Instead of forming a hyphal coil as in the wild‐type (Fig. 1b), mutants penetrated with thickened infection pegs. Subsequent colonization of the cortex resulted in the formation of arbuscules in the wild‐type (Fig. 1b), whereas all three mutants showed similar unstructured, profusely growing hyphal material (Fig. 1c–e).

To quantitatively assess the mutant phenotype we performed nurse plant inoculation (nearby growing mycorrhizal wild‐type plants), which results in efficient colonization of *vpy* mutants, while the characteristic mutant phenotype is maintained (Sekhara Reddy *et al*., 2007). All three mutants exhibited comparable amounts of colonization by extraradical hyphae, hyphopodia and intraradical hyphae, while the formation of infection coils and arbuscules was severely inhibited (Fig. S1). Instead of arbuscules, either aborted structures or abnormally retarded arbuscules were formed, and vesicle formation was reduced (Fig. S1). The similarity of the mutant phenotypes of the three *vpy* alleles suggest that they all represent functional null alleles.

### *vpy mutants exhibit cell wall alterations at sites of mycorrhizal infection*

**Fig. 2:**
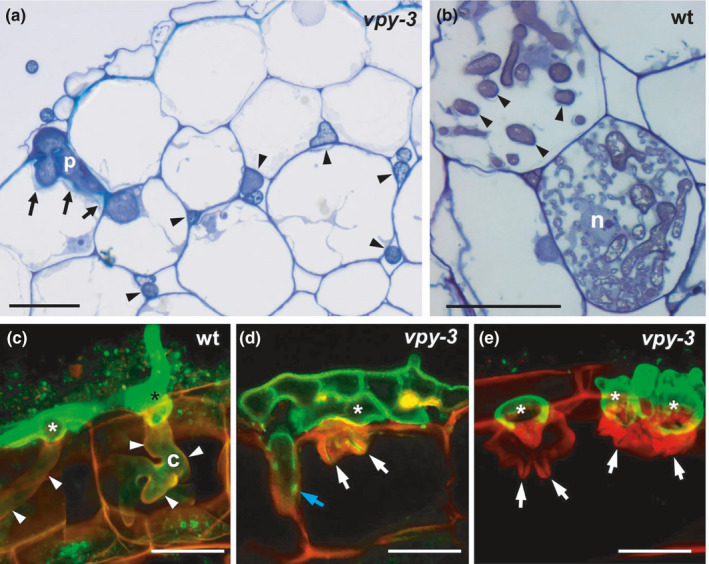
Cell wall appositions in *vpy‐3* mutant of *Petunia hybrida* infected with *Rhizophagus irregularis*. (a) Colonization pattern in *vpy‐3* mutant revealed by semithin sections of resin‐embedded material stained with toluidine blue. A hyphopodium with an intercellular infection peg (p), and cell wall appositions (arrows). Fungal colonization is mostly restricted to the intercellular spaces (arrowheads). (b) Colonization of the wild‐type (wt). Cortical cells contain abundant intracellular hyphae (arrowheads) and finely branched arbuscules, with the host nucleus (n) at a central position. (c–e) Confocal scanning micrographs of inoculated root hypodermal cells with the fungus stained by fluorescein isothiocyanate‐labeled wheat germ‐agglutinin (FITC‐WGA, green), and the plant cell walls by propidium iodide (PI, red). (c) In a wild‐type root, two fungal hyphopodia (asterisks) successfully invaded two adjacent hypodermal cells with hyphal coils. The infection hyphae are surrounded by a thin layer of plant extracellular matrix material (arrowheads). (d) On a *vpy‐3* mutant plant, the fungus has formed a highly septate complex hyphopodium (asterisk) from which it has attempted to penetrate a hypodermal cell. Thick cell wall appositions surround the fungal penetration hyphae (white arrows). A penetration hypha has inserted itself between two adjacent cells (blue arrow). (e) Extreme case of an inoculated *vpy‐3* mutant as in (d) with bloated hyphopodia (asterisks) and massive cell wall appositions (arrows) that have completely blocked fungal penetration. c, hyphal coil; p, penetration peg; n, nucleus; Bars, 40 µm.

As all three alleles exhibited a similar mutant phenotype, we selected one (*vpy‐3*) for detailed microscopic characterization. Semithin sections of resin‐embedded mycorrhizal *vpy‐3* mutants revealed that fungal hyphae were mostly confined to the apoplast at all stages of infection from the rhizodermis to the cortex (Fig. 2a, arrowheads), whereas the wild‐type showed extensive intracellular colonization by hyphal coils and arbuscules (Fig. 2b). Interestingly, hypodermal cells adjacent to fungal penetration hyphae exhibited thickened cell walls in *vpy‐3* (Fig. 2a, arrows). Confocal microscopic analysis showed that in wild‐type plants, the fungus formed hyphopodia on the root surface, from which it invaded hypodermal cells. Intracellular hyphae were surrounded by a thin layer of interfacial material (Fig. 2c, arrowheads). By contrast, hyphopodia on *vpy‐3* mutants were enlarged and septate (Fig. 2d,e). When fungal infection hyphae in the mutant produced projections into hypodermal cells, they remained short and were surrounded by thick cell wall appositions from the host (Fig. 2d,e, arrows). Occasionally, penetration proceeded between hypodermal cells in the apoplastic space (Fig. 2d, blue arrow; cf. Fig. 2a).

A second experiment confirmed that in all three *vpy* alleles, similar cell wall appositions were induced by *R*.* irregularis*. Formation of local cell wall appositions that resembled papillae (Chowdhury *et al*., 2014) was particularly strong in hypodermal cells (Fig. S2b–d), whereas the wild‐type hypodermal cells allowed invasion and growth of infection hyphae without restriction (Fig. S2a). Quantification showed that the relative number of papillae was similar in all three *vpy* alleles, whereas papillae were almost never observed in the wild‐type (Fig. S2e).

### Fungal passage through the hypodermis is blocked in *vpy‐3*

**Fig. 3:**
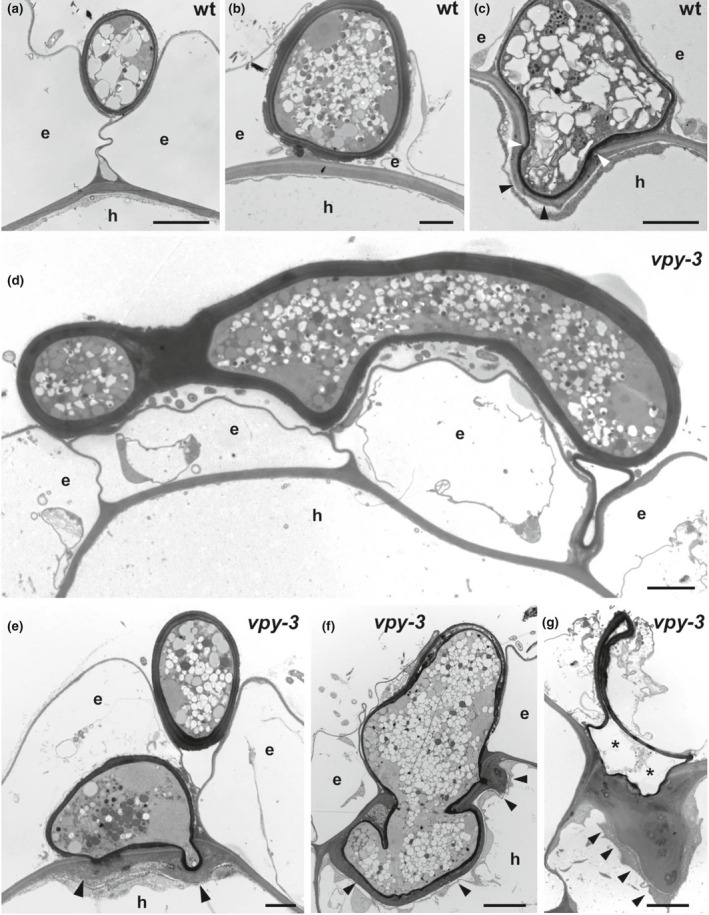
Ultrastructural analysis of root penetration in *Petunia hybrida* wild‐type and *vpy‐3*. Transmission electron micrographs of transverse sections of wildtype (wt) (a–c) and *vpy‐3* mutants (d–g) inoculated with *Rhizophagus irregularis*. All fungal structures are characterized by an electron‐dense cell wall that appears almost black. (a) Fungal hypha growing in the cleft between adjacent epidermal root cells. (b) A thick‐walled hyphopodium formed above a hypodermal cell (h) between two adjacent epidermal cells. (c) Hyphal entry point into a hypodermal cell (h). Electron translucent layers of the original cell wall were breached by the arbuscular mycorrhizal fungus (white arrowheads). The infection hypha is surrounded by a plant‐derived layer of cell wall matrix material (black arrowheads). (d) Fungal hypha growing on the root surface of *vpy‐3* and attempting to insert between adjacent epidermal cells. (e) Extraradical hypha inserted between two adjacent epidermal cells of *vpy‐3* (top), and hyphopodium‐like structure infecting a subtending hypodermal cell (h). At the entry points, the host has deposited thick layers of cell wall material (arrowheads). (f) Attempted entry into a hypodermal cell results in a distorted hyphal clump that is surrounded by an electron‐dense layer of cell wall with thickened regions (arrowheads). (g) Aborted entry in a *vpy‐3* mutant with an extremely thick cell wall papilla at the site of attempted penetration (arrowheads). The fungal hyphopodium (asterisks) has collapsed and lost most of its content. e, epidermal cell; h, hypodermal cell; Bars, 2 µm.

To assess in more detail the cellular aspects of the early stages of infection, we performed TEM on mycorrhizal wild‐type and *vpy‐3*. In the wild‐type, *R*. *irregularis* hyphae grew along the furrows between adjacent epidermal cells (Fig. 3a), followed by formation of hyphal swellings between adjacent epidermal cells (Fig. 3b). These represent the hyphopodia, from which the hypodermal cells were invaded, This process involved the breaching of external layers of the wall (Fig. 3c, white arrowheads), while the interior layers of the cell wall extended along the infection hypha to produce a continuous apoplastic sleeve that contained the fungal infection hypha (Fig. 3c, black arrowheads) (Rich *et al*., 2014). On *vpy‐3* mutant roots, *R*.* irregularis* also attempted to insert between adjacent epidermal cells (Fig. 3d), and produced structures that resembled hyphopodia; however, they appeared more irregular than hyphopodia in the wild‐type, consistent with the previously observed fungal structures revealed by light microscopy (Fig. 1,2). After forcing their way to the surface of hypodermal cells, fungal hyphae penetrated the hypodermal cell wall in a fashion that superficially resembled the invasion of wild‐type cells (compare Fig. 3c and Fig. 3e), as, in both cases, the outer layer of the cell wall (slightly more electron‐translucent) was breached, and the hyphae entered the cellular lumen. However, in contrast to the wild‐type, the mutant roots produced thick wall appositions that surrounded the inserting hyphae (Fig. 3e, arrowheads). Subsequent stages of cell invasion appeared to face resistance, as the fungal hyphae grew rather irregularly, instead of forming a typical hyphal coil (Fig. 3f). Progressive deposition of electron‐dense cell wall material around the fungus continued (Fig. 3f, arrowheads), and occasionally resulted in extreme cell wall masses (Fig. 3g, arrowheads), while the fungal structures appeared collapsed and devoid of cytoplasm, indicating that the fungus was dead (Fig. 3g, asterisks).

### AM fungal penetration triggers papilla‐like cell wall appositions in the cortex of *vpy‐3*

As *vpy* mutants have a distinct arbuscule phenotype (Figs 1c–e, 2a) (Sekhara Reddy *et al*., 2007), we assessed the AM fungal colonization pattern in the cortex of *vpy‐3* by TEM analysis. In wild‐type plants, penetration hyphae entered cortical cells by breaching the cell wall as in the case of hypodermal cells (Fig. S3a, white arrowheads), and a thin layer of interfacial cell wall material from the host was deposited on the fungal cell walls (Fig. S3a, black arrowheads). Intracellular structures of arbuscules, such as trunk hypha (TH) and fine branches (asterisks), were surrounded by a periarbuscular membrane, but hardly any interfacial cell wall material was observed between the membrane and the fungal cell wall (Fig. S3a,b). In *vpy‐3* mutants, by contrast, attempted cell penetration elicited the formation of local papillae that prevented hyphal entry (Fig. S3c). Confocal microscopy confirmed that *vpy‐3* mutants formed papillae around fungal structures, in contrast to wild‐type cells with arbuscules (Fig. S3d,e).

### Infection of *vpy* mutants by *R*. *irregularis* does not trigger accumulation of ROS

**Fig. 4:**
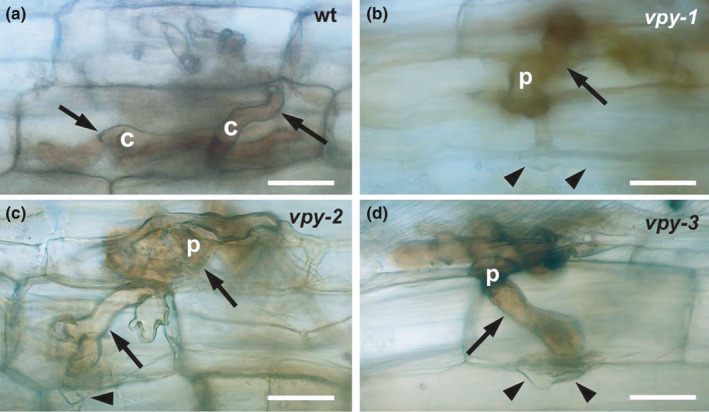
Cytochemical detection of H_2_O_2_ during infection of *vpy* mutants in *Petunia hybrida*. Mycorrhizal infection of hypodermal cells in the wild‐type (a), *vpy‐1* (b), *vpy‐2* (c) and *vpy‐3* (d) were evaluated after staining with diaminobenzidine (DAB). Strongest staining was observed in the fungal cytoplasm (arrows) of hyphal coils and infection pegs. By contrast, host cell walls and cell wall appositions adjacent to penetration hyphae (arrowheads) exhibited only weak background signal. c, hyphal coil; p, penetration peg. Bars, 25 µm.

In order to visualize H_2_O_2_ accumulation, we used 3,3′‐diaminobenzidine (DAB) staining (Daudi & O’Brien, 2016). As confocal and TEM analysis had revealed the strongest defence reactions in hypodermal cells, we focused on this cell type. Wild‐type hypodermal cells with an infection hypha showed strongest DAB signal in the hyphae, rather than in the host cytoplasm (Fig. 4a). Similarly, penetrated *vpy* mutants exhibited the strongest DAB staining inside the fungal hyphae (Fig. 4b–d), and cell wall appositions of the host did not exhibit elevated signal (Fig. 4b–d, arrowheads), suggesting that the cellular defence response in *vpy* mutants does not involve H_2_O_2_ accumulation. Further examination of cortical colonization revealed that also in the later stages of AM development, strongest DAB signal was associated with fungal structures, including arbuscules (Fig. S4b,d,e) and vesicles (Fig. S4c,f). While the staining was comparable in vesicles of *vpy‐2* and wild‐type roots (compare Figs. S4c and S4f), it was consistently weaker in cells with abnormal arbuscules in mutants vs fully developed arbuscules in the wild‐type (compare Figs S4b and S4d). This staining pattern was consistent in all three *vpy* alleles.

Next, we performed NBT staining (Kumar *et al*., 2014) to reveal O_2_^–^ accumulation. In general, NBT staining produced very strong signal in root tips of wild‐type as well as mutant roots, irrespective of their mycorrhizal status (Fig. S5a–e). Considering infection sites in colonized wild‐type roots, NBT staining was essentially restricted to fungal structures (Fig. S5f). Similarly, infected hypodermal and cortical cells in *vpy* mutants showed NBT staining almost exclusively in fungal hyphae (Fig. S5g–j). Taken together, it appears that ROS concentrations are higher in the fungal cytoplasm than in the host (except for the root tips), and that infected cells of *vpy* mutants did not accumulate higher ROS concentrations compared with the corresponding wild‐type cells.

### Abortion of fungal entry in *vpy* mutants does not correlate with callose deposition

We next tested whether papillae in *vpy* mutants contain callose, which is often associated with cellular defence (Chowdhury *et al*., 2016). Inoculated wild‐type plants did not show any sign of local callose deposition (Fig. S6a,b). Likewise, colonized *vpy‐3* mutants in general showed no callose (71%, *n* = 17) (Fig. S6c,d). However, in 12% of *vpy‐3* plants, weak callose accumulation was observed in hypodermal cells with infection hyphae (Fig. S6e,f), and in 17% of the cases, strong callose accumulation was associated with fungal structures (Fig. S6g,h). In order to further examine whether callose deposition is related to particular AM fungal fates in the mutants, we performed a second experiment in which all three mutant alleles and the wild‐type were scored for callose deposition. Wild‐type plants exhibited weak background staining (4.1%; Table S1). Similarly, *vpy* mutants showed weak spurious callose signals in 1.8–10.1% of the examined infected cells (Table S1), and a strong callose signal was observed in only three cases (Table S1). These cases did not correlate with particular deformations in the fungus (data not shown), suggesting that there is no correlation between callose formation and fungal abortion.

### Abortion of fungal penetration in petunia *vpy* mutants correlates with local lignin accumulation and induction of lignin biosynthetic genes

**Fig. 5:**
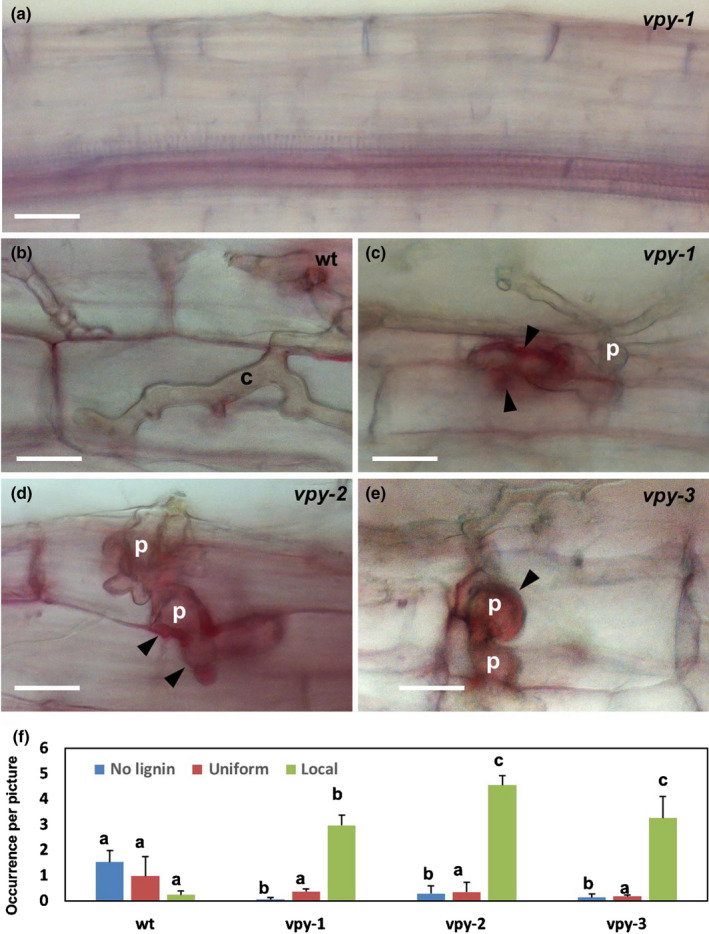
Lignin accumulation in infected hypodermal cells of *vpy* mutants in *Petunia hybrida*. Mycorrhizal roots were stained with phloroglucinol‐HCl, and entry points in hypodermal cells of wild‐type (b), *vpy‐1* (c), *vpy‐2* (d) and *vpy‐3* (e) were evaluated for lignin accumulation. (a) Nonmycorrhizal *vpy‐1* mutant root exhibits weak general signal in all cell types, and elevated signal in the central stele with the vasculature. (b–e) Infected cells of wild‐type (b), *vpy‐1* (c), *vpy‐2* (d) and *vpy‐3* (e) exhibit weak background signal in the wild‐type (b) and strong lignin accumulation (arrowheads) in cell wall appositions of *vpy* mutants (c–e). (f) Quantification of lignin accumulation reveals a correlation of strong local lignin accumulation with abortion of arbuscular mycorrhizal fungal penetration in hypodermal cells of *vpy* mutants. Data are means + SD. Different letters indicate significant differences (one‐way ANOVA, n = 3). c, hyphal coil; p, penetration peg. Bars, 50 µm in (a), 25 µm in (b‐e).

We further tested by phloroglucinol staining whether the papillae triggered by AM fungal penetration in *vpy* mutants contained lignin. In noninoculated wild‐type and mutant roots, a weak general signal in all cell types was observed, except for the stele that showed higher lignin staining (Fig. 5a). Penetrated wild‐type cells occasionally had a weak general signal, but in most cases, no accumulation over background levels was observed (Fig. 5b). By contrast, aborted infections in *vpy‐1*, *vpy‐2*, and *vpy‐3* showed strong local accumulation of lignin in cell wall appositions of the host next to the fungal entry site (Fig. 5c–e, arrowheads). Quantification revealed that all three mutant alleles exhibited a significant increase in local lignin accumulation at fungal penetration sites (Fig. 5f). For comparison, we evaluated whether the *ram1* mutant also accumulated lignin upon AM fungal infection (Fig. S7). Apart from the lignin signal in the vasculature of the stele (Fig. S7a,b), infection of the wild‐type did not lead to appreciable accumulation of lignin around fungal infection coils (Fig. S7c,d) or in cells with arbuscules (Fig. S7e,f). Similarly, the infection coils and the defective arbuscules in *ram1* were not accompanied by accumulation of lignin (Fig. S7g,h; > 30 infection and colonization sites assessed, respectively).

Lignin biosynthesis involves a well characterized biosynthetic pathway (Vanholme *et al*., 2019). Thus, we identified the respective petunia homologues for all core biosynthetic genes (Table S2), and determined their expression during AM development in the wild‐type and the *vpy* mutants by qRT‐PCR in all three *vpy* alleles. As expected from lignin stainings (Fig. 5), mycorrhizal *vpy* mutants showed a concerted induction of all lignin biosynthetic genes (Tables 1, S3–S5; Fig. S8).

**Table 1:** Expression ratios of lignin‐related genes in *Petunia hybrida* wild‐type (wt) and *vpy* mutants relative to the respective nonmycorrhizal controls (c).

	AM (wt)/c (wt)		AM (mutant)/c (mutant)		
	wt*	wt	vpy1*	*vpy2*	*vpy3*
PAL‐1	1.03	1.19	**2.00**	**5.77**	**6.80**
PAL‐2	**2.55**	**2.07**	**5.11**	**8.01**	**12.09**
PAL‐3	1.27	1.34	**4.59**	**8.39**	**5.38**
4CL‐1	**2.87**	**1.85**	**15.72**	**7.59**	**11.39**
4CL‐2	1.81	**1.40**	**9.42**	**4.73**	**7.58**
4CL‐3	0.94	1.09	**14.97**	**20.22**	**33.55**
4CL‐4	0.81	0.72	**5.49**	**15.64**	**11.57**
CA4H	0.96	1.05	**4.20**	**5.98**	**4.08**
C3H‐1	**1.52**	1.20	**7.61**	**6.75**	**7.50**
C3H‐2	**1.83**	1.03	**6.24**	**3.26**	**5.27**
COMT	1.08	**1.92**	**3.52**	**13.44**	**15.71**
CCoAOMT‐1	1.02	1.54	**6.95**	**6.74**	**6.52**
CCoAOMT‐2	1.19	1.20	**3.63**	**5.86**	**8.63**
CCoAOMT‐3	1.65	1.06	**5.29**	**18.68**	**40.17**
HCT	**2.38**	**3.39**	**5.76**	**19.76**	**41.14**
CAD1	1.23	**2.25**	**6.87**	**14.84**	**14.03**
CAD2	1.05	1.11	**8.63**	**16.76**	**29.45**
CCR1	1.17	1.49	**7.22**	**31.70**	**44.90**
CCR2	0.80	1.22	**4.97**	**8.10**	**7.08**
F5H2	1.02	1.04	**4.39**	**4.98**	**14.98**

Induction of lignin biosynthetic genes was determined by quantitative real‐time reverse‐transcriptase polymerase chain reaction with actin and glyceraldehyde‐3‐phosphate dehydrogenase (GAPDH) as reference genes. Values represent induction ratios (‐fold) derived by dividing the expression values of mycorrhizal plants (AM) by values of nonmycorrhizal controls (both normalized with the two reference genes) in wt, *vpy‐1*, *vpy‐2* and *vpy‐3* plants. All expression values were derived from six biological replicates. Color shading represents induction > two‐fold (yellow), > four‐fold (orange), and > eight‐fold (red). Data represent two independent experiments, one with only *vpy‐1* vs wild‐type (asterisks), and one with *vpy‐2* and *vpy‐3* vs wild‐type. Significant induction ratios are indicated in bold font (one‐way ANOVA, *n* = 6). Two‐way ANOVA revealed significant interactions between plant genotype and mycorrhizal treatments (see Supporting Information Table S13).

In order to test how general the lignin response in petunia *vpy* mutants is, we performed lignin staining in *sym* mutants of *M. truncatula* (Table S6; Figs S9–S11; Notes S1) (Endre *et al*., 2002; Lévy *et al*., 2004; Mitra *et al*., 2004; Kalo *et al*., 2005; Maillet *et al*., 2011; Gobbato *et al*., 2012). However, neither *vpy‐2* nor any other mutant (*ram1‐1*, *dmi2‐1*, *dmi3‐1*, *nsp2‐2*) showed lignin accumulation (Fig. S11; Notes S1), suggesting that lignin does not play a role in the repression of AM fungi in these *Medicago* mutants.

### Resistance of *vpy‐3* does not correlate with accumulation of SA, JA or ethylene

We next tested whether papilla formation in mycorrhizal *vpy* mutants is accompanied by accumulation of the defence hormones SA, JA or ethylene. In order to assess early infection events, as well as fully established mycorrhizal colonization, we tested *vpy‐3* mutant roots at 10 and 35 d after nurse plant inoculation. In general, SA concentrations were low and did not significantly change during AM infection either in the wild‐type or in the mutants (Fig. S12a). Next, we compared the concentrations of free and conjugated SA. Conjugated SA occurred in almost 10‐fold higher concentrations than free SA, but its concentrations were not induced in the mutant, independent of its mycorrhizal status (Fig. S12b). Ethylene was produced only in trace amounts in wild‐type and *vpy* mutants, and its concentrations did not change in mutants or in the wild‐type upon AM inoculation (data not shown). JA and JA‐Ile concentrations were slightly induced in wild‐type plants at the second time point, while the concentrations in mutants were not significantly changed (Fig. S13). As in the case of SA, the JA (Fig. S13a) and JA‐Ile (Fig. S13b) concentrations were generally low. Taken together, these results suggest that the cellular resistance reaction in *vpy‐3* does not involve the accumulation of SA, ethylene or JA.

### Induction of PR gene homologues in mycorrhizal roots of wild‐type and *vpy* mutants

In order to systematically assess the expression of PR genes during mycorrhizal development, we identified PR genes in petunia (Table S7) by protein blasts using established PR protein sequences (mostly from tobacco) as queries (van Loon *et al*., 2006b), and assessed their expression patterns by qRT‐PCR analysis in all three *vpy* alleles. Consistent with previous findings (Salzer *et al*., 2000; Breuillin *et al*., 2010; Campos‐Soriano *et al*., 2010), several PR gene homologues were induced in mycorrhizal roots of wild‐type plants (Tables 2, S10, S11), in particular β‐1,3‐glucanase (PR2a), chitinase (PR4b and PR4d), a thaumatin‐like gene (PR5a), a proteinase inhibitor (PR6a), a proteinase (PR7), a peroxidase (PR9) and a lipid transfer protein (PR14). In *vpy* mutants, expression of several genes was further induced (Table 2), while some were induced only in the mutants, namely PR2b (β‐1,3‐glucanase), PR3 (chitinase), PR4c (chitinase) and PR17 (unknown function). Comparing mycorrhizal mutants with mycorrhizal wild‐type plants directly showed which genes are particularly induced in the mutants (PR2a, PR2b, PR4c; Table S8, left), and comparison of nonmycorrhizal mutants and wild‐type showed that the mutants had constitutively higher levels of PR4d and PR6a expression (Table S8, right).

**Table 2:** Expression ratios of pathogenesis‐related (PR) genes in *Petunia hybrida* wild‐type (wt) and *vpy* mutants relative to the respective nonmycorrhizal controls (c).

	AM (wt)/c (wt)		AM (mutant)/c (mutant)		
	wt*	wt	vpy1*	*vpy2*	*vpy3*
PR2a	1.27	**2.53**	**18.30**	**30.47**	**13.20**
PR2b	2.02	1.42	**8.42**	**17.47**	**24.69**
PR2c	1.48	1.26	1.87	1.09	0.88
PR3	1.17	1.39	**6.58**	**5.02**	**3.11**
PR4b	2.04	**5.45**	**9.64**	**41.73**	**13.02**
PR4c	0.92	1.10	**8.71**	**10.76**	**13.82**
PR4d	**5.57**	**5.51**	**2.39**	**2.68**	**1.62**
PR5a	**6.67**	**3.48**	1.14	1.02	1.13
PR6a	1.80	13.24	6.11	**33.76**	1.57
PR7	**4.28**	**5.21**	**26.91**	**33.59**	**7.05**
PR9	**6.93**	**5.23**	**24.39**	**6.40**	**26.00**
PR14	**2.53**	**3.02**	**14.91**	**21.59**	**19.79**
PR17	1.35	1.40	**9.02**	**8.65**	**2.47**

Induction of PR genes was determined by quantitative real‐time reverse‐transcriptase polymerase chain reaction with actin and glyceraldehyde‐3‐phosphate dehydrogenase (GAPDH) as reference genes. Values represent induction ratios (‐fold) derived by dividing the expression values of mycorrhizal plants (AM) by values of nonmycorrhizal controls (both normalized with the two reference genes) in wt, *vpy‐1*, *vpy‐2* and *vpy‐3*. All expression values were derived from six biological replicates. Color shading represents induction> two‐fold (yellow), > four‐fold (orange) and> eight‐fold (red). Data represent two independent experiments, one with only *vpy‐1* vs wild‐type (asterisks), and one with *vpy‐2* and *vpy‐3* vs wild‐type. Significant induction ratios are indicated in bold (one‐way ANOVA, *n* = 6). Two‐way ANOVA revealed significant interactions between plant genotype and mycorrhizal treatments (see Supporting Information Table S13).

In order to identify PR genes that are responsive to MAMPs, we treated nonmycorrhizal wild‐type plants with a chitin hydrolysate that contains N‐acetyl‐glucosamine oligosaccharides (Chit), and an aqueous extract from *Penicillium chrysogenum* (Pen). Both preparations had previously been shown to induce a strong defence response and disease resistance in *Arabidopsis thaliana* (Thuerig *et al*., 2005). Several PR genes were induced by Pen elicitor, and only two of them were responsive to chitin (Tables S9, S12). The strongest induction was found for PR4d, one of the two genes that is constitutively induced in *vpy* mutants (Table S8).

Taken together, these results indicate that: wild‐type mycorrhizal roots express several PR genes at induced levels; mycorrhizal *vpy* mutants are overresponsive with regard to AM‐inducible PR genes, and they induce additional PR genes; and several PR genes are responsive to MAMPS in elicitor preparations.

### Cell wall appositions in *vpy‐3* contain β‐1,3‐glucanase

Pathogenesis‐related proteins may accumulate locally at sites of microbial infection, or they could be generally induced without a particular pattern of accumulation. As β‐1,3‐glucanase was induced in *vpy* mutants (PR2a and PR2b in Table 2, S8), we investigated whether papillae in *vpy* mutants contain β‐1,3‐glucanase by employing a polyclonal antiserum raised against tobacco β‐1,3‐glucanase (Beffa *et al*., 1993). Immunocytochemical detection with gold‐coupled secondary antibodies revealed a low general signal in the cell walls of noninoculated control roots in the wild‐type, as in *vpy‐3* mutants (Fig. S14a,c,e,g). Inoculated wild‐type plants did not show an obvious increase (Fig. S14b,f), consistent with the weak induction of PR2 in mycorrhizal wild‐type plants (Table 2). Inoculated *vpy‐3* mutants exhibited a general distribution of gold particles in a similar manner as the other treatments (Fig. S14d,h). However, papillae in the vicinity of aborted fungal hyphae exhibited a more prominent signal (Figs S15, S16). Hence, accumulation of β‐1,3‐glucanase appears to increase in papillae of *vpy‐3*. Quantification of immunogold signal confirmed the local accumulation of β‐1,3‐glucanase in papillae (Fig. S17).

## Discussion

### Signalling in symbiosis and defence

Transcript profiling of AM in various host species has shown that besides the induction of many AM‐related genes with presumed functions in symbiosis (e.g. phosphate and ammonium transporters), many AM‐induced genes are related to established defence genes (e.g. PR genes) (Gao *et al*., 2004; Grunwald *et al*., 2004; Deguchi *et al*., 2007; Liu *et al*., 2007; Siciliano *et al*., 2007; Fiorilli *et al*., 2009; Breuillin *et al*., 2010; Campos‐Soriano *et al*., 2010; Gaude *et al*., 2012; Handa *et al*., 2015; Fiorilli *et al*., 2018). In addition, the complex signalling mechanisms in symbiosis include, besides dedicated symbiosis signals (myc factors), molecular species that are known also to play a role in disease resistance (e.g. chitin oligomers; Zipfel & Oldroyd, 2017). Consistent with these findings, some receptors for chitinous signalling molecules have a dual role in disease resistance and symbiosis (Zipfel & Oldroyd, 2017). These findings point to overlapping mechanisms in the signalling pathways in symbiosis and defence, including for example the production of ROS (Scheler *et al*., 2013; Damiani *et al*., 2016), the induction of JA (Wasternack & Hause, 2013) and calcium‐related signals (Aldon *et al*., 2018), which all have been observed in both symbiosis and defence. Moreover, the outcome of AM symbiosis for the host plant is highly context‐dependent (Klironomos, 2003; Smith *et al*., 2009; Lanfranco *et al*., 2018). This implies that some additional mechanisms may be required to specify and determine whether roots engage in defence or in symbiosis. It is conceivable that the common symbiosis signaling pathway (CSSP) is one of the central elements to ensure that symbiosis‐related signalling overrides defence signalling during mycorrhizal infection. In addition to specific symbiosis signals, AM fungi use effectors to help prevent the induction of defence responses or to dampen their amplitude (Kloppholz *et al*., 2011; Sedzielewska Toro & Brachmann, 2016; Tang *et al*., 2016; Kamel *et al*., 2017; Chen, ECH *et al*., 2018; Voss *et al*., 2018; Morin *et al*., 2019).

### Symbiosis mutants show symptoms of a defence response

Consistent with the assumption that commitment to symbiosis requires repression of defence reactions, many mutants that are defective in symbiosis signalling (*sym* mutants) show various symptoms of a cellular defence response (Gollotte *et al*., 1993; Gianinazzi‐Pearson, 1996; Wegel *et al*., 1998; Ruiz‐Lozano *et al*., 1999; Bonfante *et al*., 2000; Marsh & Schultze, 2001; Novero *et al*., 2002; Demchenko *et al*., 2004). In such mutants, AM fungus colonization is hampered or fully blocked, cells accumulate secondary metabolites that are known (or suspected) to act as defence agents, cell walls are altered or reinforced, and defence‐related transcripts are induced. This is strong evidence that one function of symbiotic signalling is to avoid, or repress, defence during AM interactions.

In *vpy* mutants, defence‐like aspects of the AM‐defective phenotype are particularly prominent. Here, we show that this syndrome resembles a bona‐fide cellular defence response, involving the formation of cell wall papillae (Figs 2, 3, S2, S3), the local accumulation of lignin (Fig. 5), the induction of lignin‐biosynthetic genes (Table 1), and the induction of a range of PR genes (Table 2). The fact that *vpy* mutants in petunia show these symptoms of defence suggests that one function of VPY is to suppress, directly or indirectly, the induction of defence. This appears to be a prerequisite for intracellular accommodation of AM fungi during infection at the root surface, and during arbuscule development in the cortex (Ercolin & Reinhardt, 2011; Gutjahr & Parniske, 2013). An interesting aspect of the colonization phenotype of *vpy* mutants in both petunia and *Medicago* is that intercellular hyphal colonization is not inhibited (Figs 2a, S10g) (Sekhara Reddy *et al*., 2007), suggesting that the interaction at the extracellular level is compatible. A similar phenotype was observed in *della* mutants that were essentially devoid of intracellular structures yet exhibited high colonization levels by profusely growing intercellular hyphae (Floss *et al*., 2013).

### Defence response in petunia *vpy* mutants involves local lignin accumulation

Papilla formation is considered a highly effective defence mechanism of plants against fungal pathogens (Hückelhoven, 2007). Papillae inhibit fungal penetration in aerial plant tissues and often result in complete abortion of the pathogen. In the root, the formation of cell wall reinforcements as defence mechanism has been less explored. The cell wall appositions in mycorrhizal *vpy* mutants colonized by *R*. *irregularis* were either locally restricted to the site of cell penetration, or they surrounded intracellular hyphae, thereby encapsulating them (Figs 2, 3, 5, S2, S3, S15).

Interestingly, the papillae of *vpy* mutants did not, in most cases, contain callose, and they were only weakly autofluorescent, in contrast to previous reports in pea symbiosis mutant *P2* (Gianinazzi‐Pearson *et al*., 1996), indicating that some hallmarks of defence are absent in mycorrhizal *vpy* mutants. However, papillae were impregnated by local accumulation of lignin (Fig. 5). Consistent with this observation, the lignin biosynthetic pathway was induced in a concerted fashion, in inoculated *vpy* mutants, involving all biosynthetic genes (Fig. S8) (Vanholme *et al*., 2019). Cell wall lignification is among the most effective cellular defence responses of plants. In general, high lignin content correlates with increased disease resistance in various plant species (Miedes *et al*., 2014). For example, lignin contributes to penetration resistance of wheat against powdery mildew (Bhuiyan *et al*., 2009). In tomato, roots of a cultivar resistant to the pathogen *Ralstonia solanacearum* accumulate higher lignin concentrations than a susceptible cultivar (Mandal *et al*., 2011). Whether the lignin was confined to papillae was not addressed in this case. It is difficult to attribute effective resistance to particular cell wall components (cellulose, hemicellulose, callose, lignin, cell wall proteins etc.), as papillae often have a complex composition. Callose is generally accepted as an important element that significantly reinforces papillae against pathogen penetration (Hückelhoven, 2014) and that is thought to contribute to MIR (Cordier *et al*., 1998); however, lignin impregnation is a rather unusual feature of papillae and has not been reported earlier in the context of resistance against AM fungi in *sym* mutants. Given the well‐documented role of lignin in reinforcing cell walls (Miedes *et al*., 2014), it is plausible to assume that lignin contributes to the abortion of AM fungal cell penetration in petunia *vpy* mutants.

In addition, the concerted induction of enzymes in the phenylpropanoid pathway (Table 1; Fig. S8) could potentially lead to the production of other defence‐related compounds (Dixon *et al*., 2002; Vogt, 2010). Interestingly, the *ram1* mutant which exhibits a later defect in AM development during arbuscule formation, and which does not display markers of a cellular defence response (Park *et al*., 2015; Rich *et al*., 2015; Pimprikar *et al*., 2016), did not accumulate lignin (Fig. S7). Strikingly, in *M*. *truncatula vpy‐2* mutants (as well as in *dmi2‐1*, *dmi3‐1*, *nsp2‐2* and *ram1‐1* mutants), no accumulation of lignin was detected, suggesting that in *M*.* truncatula* roots, abortion of AM fungal infection does not involve lignin. This points to taxon‐specific aspects in root defence, and possibly in AM‐related pathways between petunia and *M*.* truncatula*, and perhaps, more generally, between Solanaceae and legumes.

### Induction of PR genes in mycorrhizal wild‐type and *vpy* mutants

A characteristic symptom of defence in plants is the accumulation of PR proteins (van Loon *et al*., 2006b). Several PR proteins have been shown to have antimicrobial activity, and some of them contribute to disease resistance; hence they are regarded as markers of defence. We assessed the expression of all PR gene homologues that could be identified in the petunia genome (Bombarely *et al*., 2016), and 13 were found to be expressed in roots (Table 2). PR genes have previously been reported to be induced in many mycorrhizal associations (reviewed in García‐Garrido & Ocampo, 2002). In most cases, the induction was characterized as early, weak and transient (Gianinazzi‐Pearson *et al*., 1996; Kapulnik *et al*., 1996). However, in some cases, PR genes were induced in a sustained fashion and at high levels (Salzer *et al*., 2000; Breuillin *et al*., 2010), indicating that they may have an important role in AM symbiosis. In addition, the phylogenomic signature of some AM‐induced PR proteins (e.g. chitinase III) indicates that they have been under positive selection in AM‐competent plant species (Favre *et al*., 2014; Rich *et al*., 2014), suggesting that they have specific functions in AM.

We observed that several PR genes were induced in mycorrhizal wild‐type roots (Table 2), possibly reflecting an elevated defence status in mycorrhizal roots (García‐Garrido & Ocampo, 2002; Pozo & Azcon‐Aguilar, 2007). In mycorrhizal *vpy* mutant roots, several PR genes (PR2a, PR2b, PR4c, PR7, PR9, PR14 and PR17) were expressed at even higher levels than in the wild‐type, consistent with our findings that mycorrhizal *vpy* mutants mount a defence response (Figs 2, 3, 4, 5). The strong induction of two PR2 genes in *vpy* mutants, and the local accumulation in papillae of PR2 protein (Figs S15, S17) are of particular interest as transgenic tobacco‐overexpressing PR2 exhibited significantly delayed mycorrhizal colonization of the roots (Vierheilig *et al*., 1995). Hence, PR2, and perhaps other PR proteins, could be involved in restricting mycorrhizal colonization in *vpy* mutants.

### Defence response in *vpy* mutants does not affect concentrations of SA, JA, ethylene or ROS

Initiation of a defence response in plants is often associated with the accumulation of the stress and defence hormones SA, JA or ethylene (Browse, 2009; Vlot *et al*., 2009; Broekgaarden *et al*., 2015). In mycorrhizal roots, the picture is more complex, as SA and JA can be either up‐ or downregulated during symbiosis (Fernandez *et al*., 2014). We found no significant differences in the concentrations of free or conjugated SA (Fig. S12), consistent with the general lack of expression of the SA‐marker PR1 in roots. JA and JA‐Ile concentrations were not affected at the early time point of the interaction (10 d), but were later slightly induced in the wild‐type, in contrast to *vpy‐3* mutants (Fig. S13). JA concentrations are known to be induced in mycorrhizal roots in some cases; however, the role of JA in symbiosis is controversial (Wasternack & Hause, 2013).

A commonly observed phenomenon associated with plant defence is the production of ROS in the host (Torres *et al*., 2006). In AM, ROS may also play a role, although in this case, it is not clear which of the symbiotic partners is the main source of ROS (Fester & Hause, 2005). We used two established staining procedures to detect H_2_O_2_ (DAB) and O_2_^–^ (NBT), respectively. Interestingly, both methods revealed high ROS concentrations in the fungus, irrespective of the host genotype (Figs 4, S4,S5). As described in *M*. *truncatula* (Salzer *et al*., 1999), highest H_2_O_2_ concentrations were associated with clumped arbuscules that appeared to undergo senescence (Fig. S4). NBT staining in the host was strongest in root tips (Fig. S5a–e), as has been shown for *Arabidopsis* and *Medicago* (Dunand *et al*., 2007; Chen *et al*., 2015). In infected areas of the cortex, NBT signal was confined to fungal hyphae (Fig. S5f–j) and arbuscules (in the wild‐type). Taken together, these results show that none of the classical defence hormones (SA, JA, ethylene) were induced during the defence response in *vpy‐3*, and ROS accumulation patterns did not reveal a correlation with defence in *vpy* mutants.

### VAPYRIN is involved in repression of cellular defence

How could VPY interfere with defence? The localization of VPY to mobile subcellular compartments (Feddermann *et al*., 2010; Pumplin *et al*., 2010), and its interaction with EXO70I and EXO70H4 (Zhang *et al*., 2015; Liu *et al*., 2019) suggest that VPY could be involved in subcellular trafficking towards fungal hyphae. Hence, the mobile compartments could transport a cargo or a membrane component that interferes with the induction of defence. Alternatively, VPY could be a target of an AM fungal effector which represses defence in the host. Future research should identify the interaction partners of VPY and potential downstream components to explain how it acts on defence mechanisms.

## Author contributions

MC, SB, LB, GD, MS and DR planned and conducted the experiments and were involved in data collection. GG performed hormone analytics. SB, MC, and DR were involved in data analysis. DR wrote the paper with assistance from SB and MC.

## Supporting information


**Fig. S1** Quantitative phenotypic analysis of *vpy* mutants.
**Fig. S2** Confocal analysis of papilla formation in *vpy* mutants.
**Fig. S3** Infection phenotype in *vpy‐3* root cortex cells.
**Fig. S4** Cytochemical detection of H_2_O_2_ in the cortex of *vpy* mutants.
**Fig. S5** Cytochemical detection of O_2_^–^ in *vpy* mutants.
**Fig. S6** Callose accumulation associated with aborted fungal infection of *vpy‐3*.
**Fig. S7** Lack of lignin accumulation in mycorrhizal *ram1* mutants.
**Fig. S8** Concerted induction of lignin biosynthetic genes in mycorrhizal *vpy* mutants.
**Fig. S9** Mycorrhizal colonization pattern in *M*. *truncatula* *sym* mutants.
**Fig. S10** Mycorrhizal colonization pattern in *M*. *truncatula* *sym* mutants.
**Fig. S11** Lack of lignin accumulation at fungal entry points in *M*. *truncatula* *sym* mutants.
**Fig. S12** Salicylic acid concentrations in mycorrhizal wild‐type and *vpy‐3* roots.
**Fig. S13** Jasmonic acid concentrations in mycorrhizal wild‐type and *vpy‐3* roots.
**Fig. S14** Immunocytochemical analysis of β‐1,3‐glucanase in mycorrhizal roots.
**Fig. S15** Accumulation of β‐1,3‐glucanase in a cell wall apposition of *vpy‐3*.
**Fig. S16** Low‐magnification overview picture of the sample shown in Fig. S15.
**Fig. S17** Quantification of β‐1,3‐glucanase immunogold signal.
**Methods S1** Methods relating to experiments on *Medicago truncatula*, β‐1,3‐glucanase immunostaining, RNA extraction and qPCR, and statistical analysis.
**Notes S1** Characterization of lignin accumulation in *Medicago truncatula* wild‐type and symbiosis mutants.
**Table S1** Quantification of callose accumulation in *vpy* mutants.
**Table S2** Primers used for qRT‐PCR of lignin‐related genes.
**Table S3** Induction of lignin‐related genes in *vpy* mutants vs wild‐type in either the mycorrhizal or nonmycorrhizal context.
**Table S4** Expression values for lignin‐related genes for the experiment on *vpy‐1* from the genes for which induction ratios were calculated in Tables 1 and S3.
**Table S5** Expression values for lignin‐related genes for the experiment on *vpy‐2* and *vpy‐3* from the genes for which induction ratios were calculated in Tables 1 and S3.
**Table S6** *M*. *truncatula* mutants used in this study.
**Table S7** Primers used for qRT‐PCR of PR genes.
**Table S8** Induction of pathogenesis‐related (PR) genes in mutants vs wild‐type in either the mycorrhizal or nonmycorrhizal context.
**Table S9** Induction of pathogenesis‐related (PR) genes in wild‐type plants treated with fungal elicitor preparations.
**Table S10** Expression values for the experiment on *vpy‐1* from the genes for which induction ratios were calculated in Tables 2 and S8.
**Table S11** Expression values for the experiment on *vpy‐2* and *vpy‐3* from the genes for which induction ratios were calculated in Tables 2 and S8.
**Table S12** Expression values from the experiment with wild‐type plants treated with elicitors from genes for which induction ratios were calculated in Table S9.

**Table S13** *P*‐values from statistical analyses and interaction report from two‐way ANOVA tests.
Please note: Wiley Blackwell are not responsible for the content or functionality of any Supporting Information supplied by the authors. Any queries (other than missing material) should be directed to the *New Phytologist* Central Office.
