# Supplementary material for: VAPYRIN attenuates defence by repressing PR gene induction and localized lignin accumulation during arbuscular mycorrhizal symbiosis of Petunia hybrida
Source: New Phytol. 2020 Dec 25;229(6):3481–96. doi: 10.1111/nph.17109 (PMC7986166; doi:10.1111/nph.17109)
Supplement: Supplementary file 1 — Fig. S1 Quantitative phenotypic analysis of vpy mutants. Fig. S2 Confocal analysis of papilla formation in vpy mutants. Fig. S3 Infection phenotype in vpy‐3 root cortex cells. Fig. S4 Cytochemical detection of H2O2 in the cortex of vpy mutants. Fig. S5 Cytochemical detection of O2 – in vpy mutants. Fig. S6 Callose accumulation associated with aborted fungal infection of vpy‐3. Fig. S7 Lack of lignin accumulation in mycorrhizal ram1 mutants. Fig. S8 Concerted induction of lignin biosynthetic genes in mycorrhizal vpy mutants. Fig. S9 Mycorrhizal colonization pattern in M. truncatula sym mutants. Fig. S10 Mycorrhizal colonization pattern in M. truncatula sym mutants. Fig. S11 Lack of lignin accumulation at fungal entry points in M. truncatula sym mutants. Fig. S12 Salicylic acid concentrations in mycorrhizal wild‐type and vpy‐3 roots. Fig. S13 Jasmonic acid concentrations in mycorrhizal wild‐type and vpy‐3 roots. Fig. S14 Immunocytochemical analysis of β‐1,3‐glucanase in mycorrhizal roots. Fig. S15 Accumulation of β‐1,3‐glucanase in a cell wall apposition of vpy‐3. Fig. S16 Low‐magnification overview picture of the sample shown in Fig. S15. Fig. S17 Quantification of β‐1,3‐glucanase immunogold signal. Methods S1 Methods relating to experiments on Medicago truncatula, β‐1,3‐glucanase immunostaining, RNA extraction and qPCR, and statistical analysis. Notes S1 Characterization of lignin accumulation in Medicago truncatula wild‐type and symbiosis mutants. Table S1 Quantification of callose accumulation in vpy mutants. Table S2 Primers used for qRT‐PCR of lignin‐related genes. Table S3 Induction of lignin‐related genes in vpy mutants vs wild‐type in either the mycorrhizal or nonmycorrhizal context. Table S4 Expression values for lignin‐related genes for the experiment on vpy‐1 from the genes for which induction ratios were calculated in Tables 1 and S3. Table S5 Expression values for lignin‐related genes for the experiment on vpy‐2 and vpy‐3 from the genes for which inducti [file NPH-229-3481-s001.pdf]

New Phytologist Supporting Information

Article title: VAPYRIN attenuates defence by repressing PR gene induction and localised lignin accumulation during arbuscular mycorrhizal symbiosis of Petunia hybrida

Authors: Min Chen, Sébastien Bruisson, Laure Bapaume, Geoffrey Darbon, Gaëtan Glauser, Martine Schorderet and Didier Reinhardt

Article acceptance date: 16 November 2020

Notes S1 - Supplementary Results

Medicago truncatula sym mutants do not accumulate lignin

In order to assess whether the lignin accumulation phenotype in petunia *vpy* mutants is related to a conserved function of VPY in suppression of defense, we tested whether *M. truncatula vpy* mutants showed a similar response as petunia *vpy* mutants. Furthermore, we included a number of additional *M. truncatula* mutants with a defect in AM symbiosis (**Table S6**), namely *dmi2-1* (defective in *SYMBIOSIS RECEPTOR-LIKE KINASE*, *SYMRK*) (Endre *et al.*, 2002), *dmi3-1* (defective in *CALCIUM AND CALMODULIN-DEPENDENT PROTEIN KINASE*, *CCaMK*) (Lévy *et al.*, 2004; Mitra *et al.*, 2004), *ram1-1* (defective in the *RAM1* orthologue of petunia *RAM1*) (Gobbato *et al.*, 2012), and *nsp2-2* (defective in *NODULATION SIGNALLING PATHWAY2*, *NSP2*) (Kalo *et al.*, 2005). As in the case of petunia, the mutants (and the wild type) were inoculated by chive nurse plants to achieve comparable levels of overall root colonization (>50% root length colonization) in all *Medicago* genotypes. Phenotypic assessment of these essentially confirmed all the symbiotic defects described previously (**Fig. S9, S10**). While the wild-type accessions A17 and R108 sustained the formation of hyphal coils in external cells (**Fig. S9a,b**), and of arbuscules in the root cortex (**Fig. S9c**), fungal penetration of the root surface was inhibited in *dmi2* and *dmi3* mutants (**Fig. S9d,f**). However, due to the strong inoculum potential of nurse plants, the fungus eventually entered the roots of both mutant genotypes. Subsequent cortical colonization was, however, markedly different: In *dmi3-1*, only intercellular hyphal colonization was observed (**Fig. S9e**), while in *dmi2-1*, arbuscules were formed at high frequency (**Fig. S9g**), as previously described (Demchenko *et al.*, 2004).

Ram1-1 and *nsp2-2* showed milder phenotypes (**Fig. S10a-d**). In both cases, hyphal coils were formed upon penetration of the root surface (**Fig. S10a,c**), however, arbuscule formation was affected in contrasting ways: *ram1-1* mutants only allowed for the formation of small ill-defined arbuscular structures (**Fig. S10b**, arrows), as described before (Park *et al.*, 2015; Rich *et al.*, 2015). In contrast, *nsp2-2* contained patchy colonization with apparently normal arbuscules interspersed with arbuscule-free regions or retarded arbuscules (**Fig. S10d**, arrows), implicating *NSP2* in both, nodulation as well as AM (Maillet *et al.*, 2011).

Of particular interest was the *Medicago vpy-2* mutant (**Fig. S10e-h**). *R. irregularis* grew profusely on the root surface and produced complex hyphopodia with many septa (**Fig. S10e**). Occasionally, roots were invaded by thick penetration pegs (**Fig. S10f**), followed by massive intercellular colonization in the cortex (**Fig. S10g**, arrows). Arbuscules were completely missing, however, intercellular hyphae produced many irregular lateral projections (**Fig. S10h**), presumably representing attempts to invade and colonize cortex cells. Taken together, this pattern of fungal colonization resembles the colonization phenotype of the petunia *vpy* mutant (Sekhara Reddy *et al.*, 2007).

We next treated colonized roots of all *Medicago* genotypes with phloroglucinol to reveal lignin deposition. As in petunia, both wild type accessions (A17 and R108) did not accumulate any lignin at the point of fungal infection (**Fig. S11a,b**). Surprisingly, however, none of the mutants accumulated significant amounts of lignin at infection points (**Fig. S11c-g**), including the *vpy-2* mutant, while prominent lignin deposition was observed along the vasculature in the stele (**Fig. S11h**), as in all other genotypes (data not shown). These results indicate that lignin accumulation at AMF infection points is not part of the phenotype of symbiosis mutants in *Medicago*.

Methods S1 - Supplementary Material and Methods

Medicago truncatula growth conditions and treatments

Seeds of *Medicago truncatula* were scarified with concentrated sulfuric acid for ten minutes, then rinsed five times with sterile water. Subsequently, seeds were surface-sterilised in concentrated Clorox for two minutes, followed by addition of an equal volume of water and an additional minute of sterilization. Then seeds were rinsed with sterile water five times and imbibed at room temp on an orbital shaker for four hours. Then seeds were placed on 0.8 % plant agar in a growth chamber (20 ºC, 18 h light) for germination. Seedlings were transferred to sterilized double plastic jars with perlite in the top and B&D nutrient solution (Broughton & Dilworth, 1971) containing 1mM KNO_3 in the bottom jar, connected by a cotton wick. After one month, seedlings were transferred to nurse plant pots with mycorrhizal jive plants that had been inoculated at least one month before with *R. irregularis*.

β-1,3-Glucanase immunostaining

Wild type an *vpy-3* plants were inoculated with nurse plant inoculum of *R. irregularis*. Root material was fixed for 2h with 2% (w/v) paraformaldehyde and 1% (v/v) glutaraldehyde at room temperature. Fixed material was dehydrated and embedded in Lowicryl K4M (www.sigmaaldrich.com) as described (Altman *et al.*, 1984) with the following modifications: embedding involved a series of lowicryl diluted with 95% (v/v) ethanol as follows: 1:2 (vol:vol) for 2h, 1:1 for 2h, 2:1 overnight, followed by twice 100% lowicryl for 2h and polymerization at 55°C. Ultrathin sections (70 nm) were blocked with 20 mM NH_4Cl, 20 mM lysine and 20 mM glycine. Rabbit antiserum raised against extracellular glucanase (Beffa *et al.*, 1993) was used at a dilution of 1:5 in blocking agent. Goat-anti-rabbit antibodies coupled to 10 nm gold beads (http://www.bbisolutions.com) served as secondary antibodies. Contrasting was performed with 2% (w/v) uranyl acetate ($UO_2(CH_3COO)_2$) and lead citrate solution prepared according to (Reynolds, 1963). Controls without the primary antibody did not show any labeling. For quantification of immunogold signal, from each treatment six representative pictures of hypodermal cells from two independent experiments were printed out at the same magnification. A 2D grid of small crosses at regular distances (1 cm) printed on a transparent foil was overlaid and the gold particles were counted in the respective cellular compartments relative to the number of crosses within the same compartment.

RNA extraction and quantitative real-time RT-PCR

For gene expression analysis, plants were either inoculated for four weeks with nurse plants (**Tables 1,2**), or wild type plants were treated with chitin oligosaccharides or with a *Penicillium* preparation (Thuerig *et al.*, 2005) for 1 and 4h at concentrations of 1 µg/ml and 10 µg/ml, respectively (**Table S9**). Frozen petunia roots were placed in 2 mL Eppendorf tubes containing a glass bead and were ground using a ball mill. Total RNA was extracted from the powdered roots according to the protocol of the Direct-zol RNA miniprep kit from Zymo Research

(https://www.zymoresearch.com), using trizol solution for lysis (38% v/v), saturated phenol (pH 8), 0.8 M guanidine thiocyanate, 0.4 M ammonium thiocyanate, 0.1M Na-acetate pH 5, and 5% (v/v) glycerol. The Direct-zol RNA kit involves a DNAse step to remove genomic DNA during RNA extraction. RNA was used for reverse transcription according to the protocol of the SensiFAST^{tm} cDNA synthesis kit from Bioline. PCR reactions were carried out with 5 μL of 100x diluted cDNA solution, 1 μL of 10 μM forward and reverse primer, 7.5 μL of SensiMix SYBR Hi-ROX (BIO-RAD) and DNAse/RNAse free water up to 15 μL. The reaction cycle was 95°C for 10 min followed by 45 amplification cycles (95°C for 20s, 64°C for 20s, 72°C for 20s). All samples were analyzed in technical duplicates from six independent replicate plants. Actin and glyceraldehyde-3-phosphate dehydrogenase (GAPDH) were used as reference genes. Relative expression values were calculated using the delta-Ct method (Pfaffl, 2001). Data are expressed as -fold changes in relative gene expression (AM/control; *vpy*/wt) in Tables 1, 2, S3, S8, and S9. Expression values in Tables S4, S5, and S10-S12 represent expression values normalized to actin and GAPDH. Gene expression data were statistically analyzed using two-way ANOVA for interaction between genotype and treatment, followed by one way ANOVA as described below.

Statistical analysis

qPCR results were tested by two-way ANOVA with host genotype and mycorrhizal status as the two factors of interest. The p-values of the two factors and their interactions are listed in **Table S13**. All other data, and the derived gene induction ratios from qPCRs, were treated by one-way ANOVA to test for significance of differences among groups (mutants vs. wild type, AM vs. controls) and the corresponding p-values are also listed in **Table S13**. Statistical analysis of callose accumulation (**Table S1**) was performed by Fisher's exact test for count data to test individual mutants vs. the wild type. This test revealed that callose deposition patterns in the three mutants were not significantly different from the wild type (see below):

```
> fisher.test(M[,c(1,2)])
Fisher's Exact Test for Count Data
data:  M[, c(1, 2)]
p-value = 0.3824
alternative hypothesis: true odds ratio is not equal to 1
95 percent confidence interval:
  0.264316   29.001559
sample estimates:
odds ratio
  2.367128
```

```
> fisher.test(M[,c(1,3)])
Fisher's Exact Test for Count Data
data:  M[, c(1, 3)]
p-value = 0.2099
alternative hypothesis: true odds ratio is not equal to 1
95 percent confidence interval:
 0.06014388   1.60903773
sample estimates:
odds ratio
 0.3666198
```

```
> fisher.test(M[,c(1,4)])
```

Fisher's Exact Test for Count Data
data: M[, c(1, 4)]
p-value = 0.7295
alternative hypothesis: true odds ratio is not equal to 1
95 percent confidence interval:
 0.1069708   3.8126996
sample estimates:
odds ratio
 0.7129047

Supplementary Figures and Tables

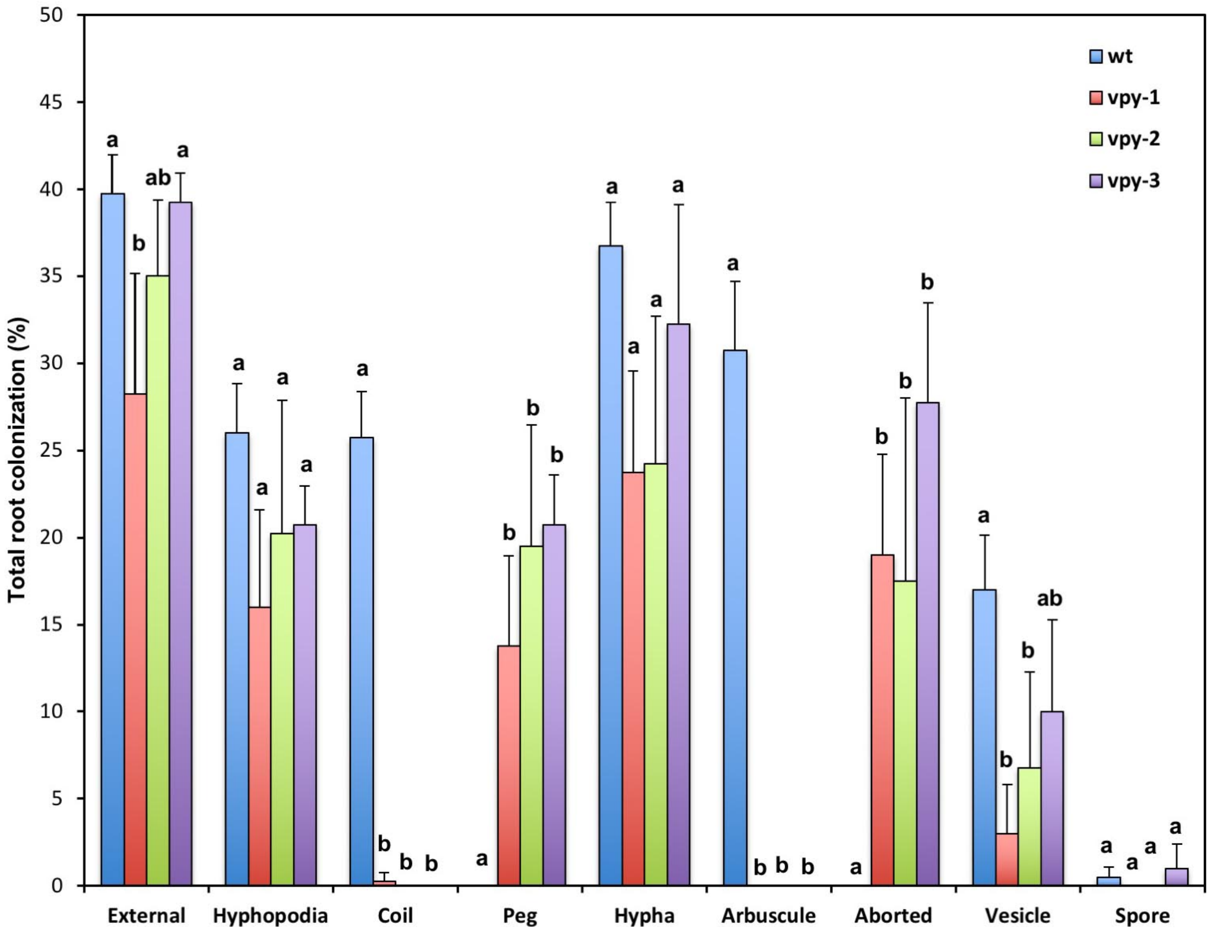

Figure S1. Quantitative phenotypic analysis of *vpy* mutants.
Mycorrhizal roots of wild type and *vpy* mutants were stained with Trypan Blue for quantitative assessment of fungal structures as indicated. Mutants formed infection pegs instead of intracellular coils in the hypodermis, and aborted or malformed structures instead of arbuscules. Shown are the mean + Stdv (n=4). Different letters indicate significant differences (one-way ANOVA).

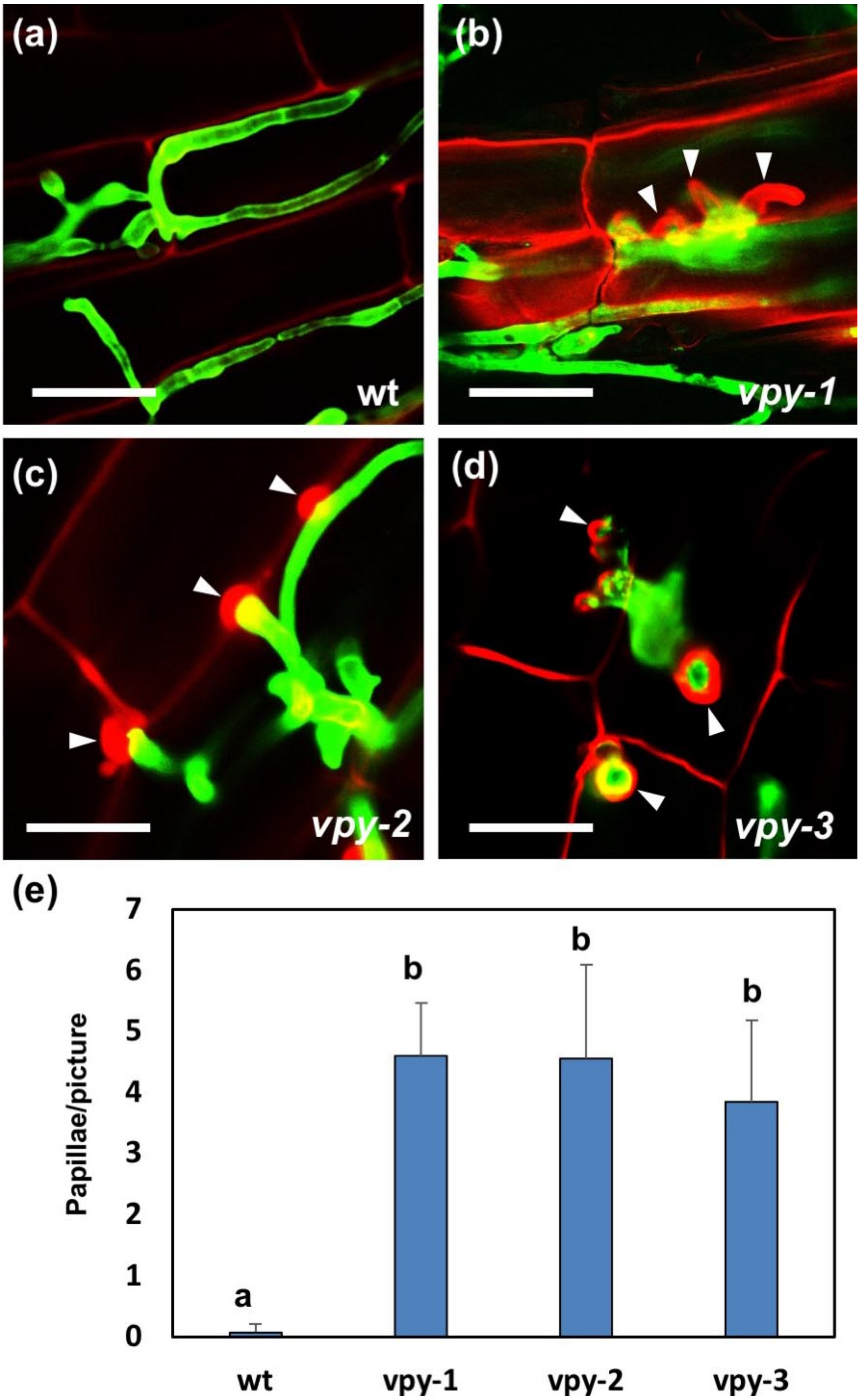

Figure S2. Confocal analysis of papilla formation in *vpy* mutants.
Mycorrhizal roots of wild type (a), *vpy-1* (b), *vpy-2* (c), and *vpy-3* (d), were stained with basic fuchsin and wheat germ agglutinine (WGA)-Alexa488, and evaluated for cell wall appositions by confocal microscopy. Shown are hyphae (green) and cell walls with papillae (red). Papillae are indicated by arrowheads. (e) Quantification of papilla formation. Representative pictures of penetrated cells as in (a)-(d) were assessed for the number of papillae. Shown are the mean + Stdv of three samples containing at least eight pictures each. Different letters indicate significant differences (one-way ANOVA, n=3). Size bar 25 µm.

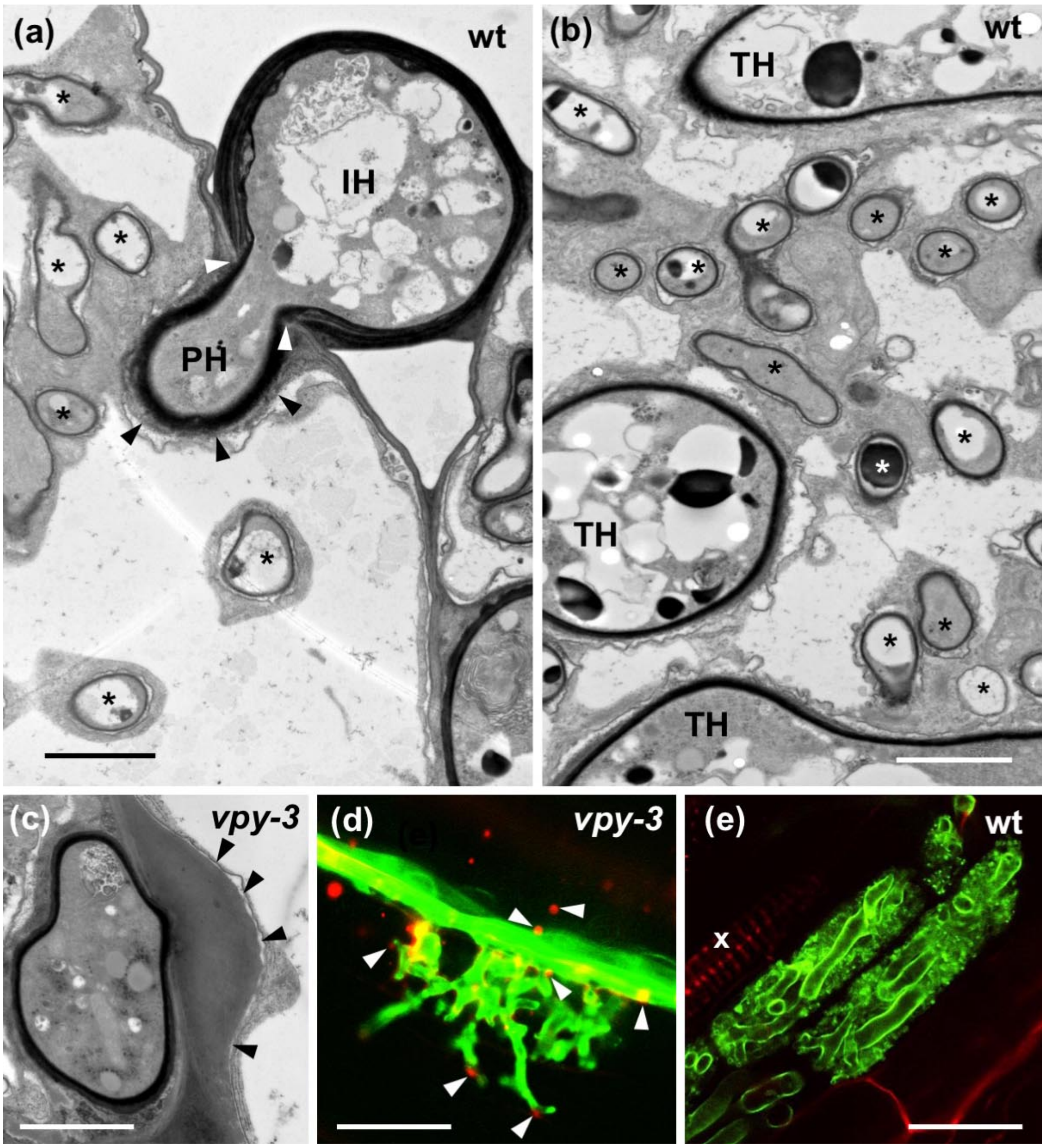

Figure S3. Infection phenotype in *vpy-3* root cortex cells.
Transmission electron micrographs of mycorrhizal root cortex cells in wild type (a,b,e), and *vpy-3* (c,d). (a) An intercellular hypha (IH) has produced an intracellular penetration hypha (PH) that has penetrated the host cell wall (white arrowheads) and that is surrounded by a thin layer of host cell wall material (arrowheads). Several thin-walled fine fungal arbuscule branches are embedded in the cytoplasm of the host (asterisks). The space between the fungal cell wall and the periarbuscular membrane of the host is very thin.
(b) Fully colonised cortex cell containing an arbuscule. Note large trunk hyphae (TH) and thin arbuscular branches (asterisks).
(c) Attempted entry into a *vpy-3* cortex cell results in formation of a papilla (arrowheads).
(d) Branched fungal structure in the cortex of a *vpy-3* root. In the vicinity of the fungal hyphae (green), multiple small papillae (red) were formed (arrowheads).
(e) Two arbuscules in the cortex of a wild type plant. Fungal hyphae (green) did not elicit any papillae.
(a)-(c) electron micrographs; (d,e) Confocal micrographs. PH, penetration hypha; TH, trunk hypha; x, xylem; IH, intercellular hypha. Scale bars in (a-c), 2 µm; in (d,e) 25 µm.

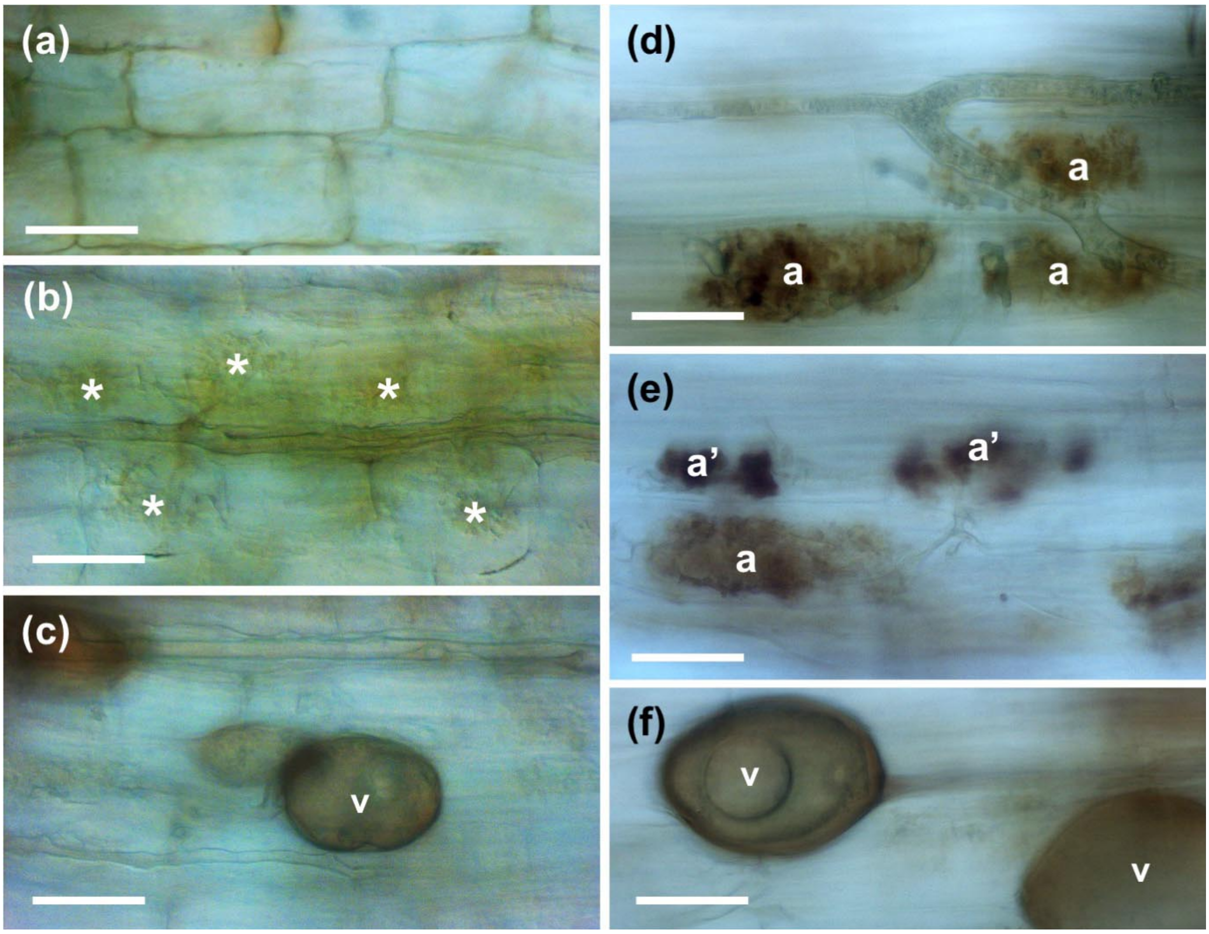

Figure S4. Cytochemical detection of H_2O_2 in the cortex of *vpy* mutants
Mycorrhizal roots of wild type and all *vpy* alleles were stained with 3,3'-diaminobenzidine (DAB). Shown are representative DAB-stained *vpy-2* mutant roots (a-c), and corresponding wild type roots (d-f). All three *vpy* alleles gave similar results.
(a) Weak background staining in *vpy-2*. The other *vpy* mutants and the wild type showed a similar background signal.
(b) Weak signal associated with residual intracellular colonisation in *vpy-2* cortex cells (a).
(c) Strong signal in vesicles (v).
(d) In wild type roots, strong staining was observed in arbuscules (a).
(e) Staining was further increased in cells with clumping arbuscules (a'), relative to mature arbuscules (a).
(f) Strong signal in vesicles (v) in the wild type cortex. Size bar 50 µm.

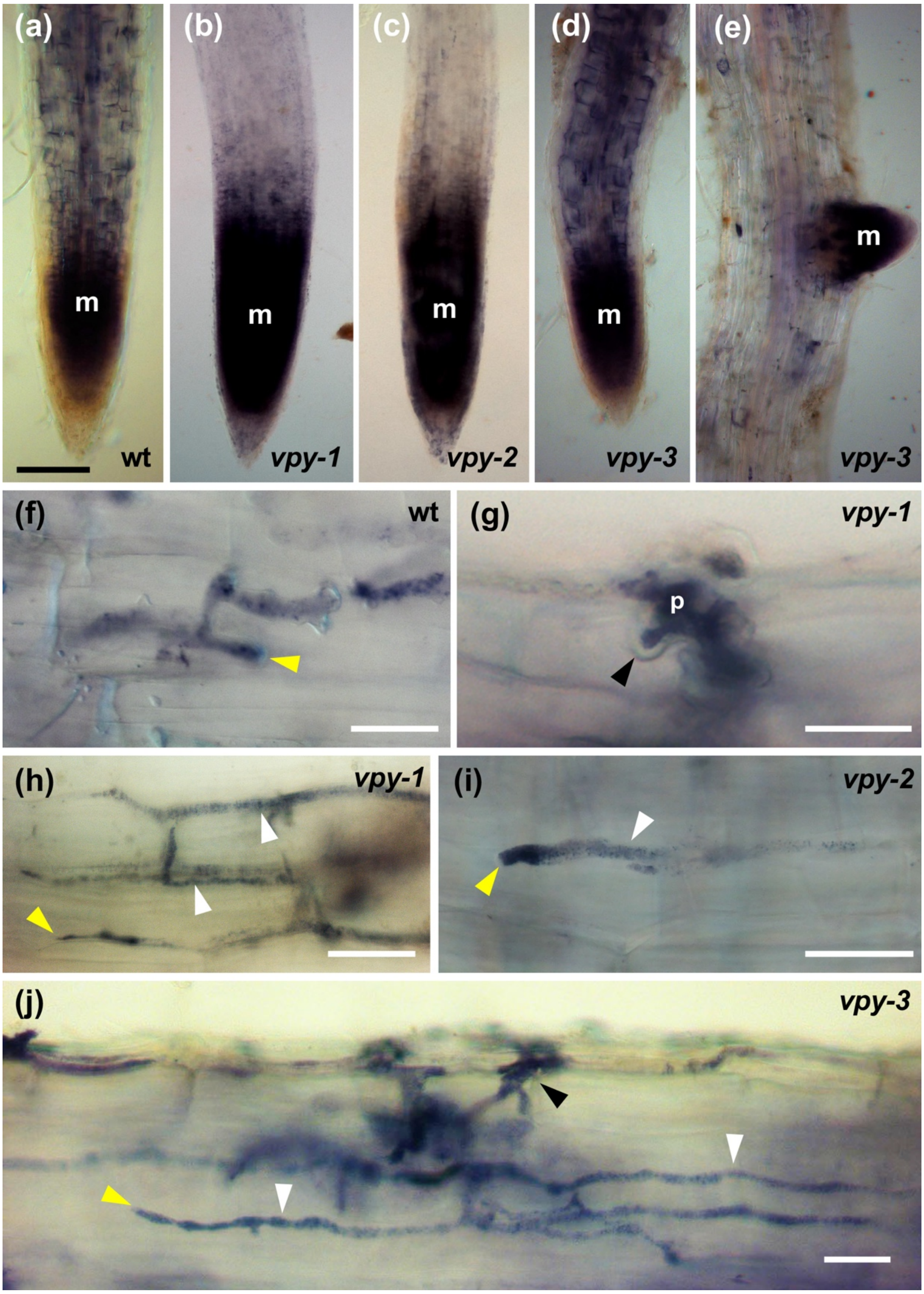

Figure S5. Cytochemical detection of O_2^- in *vpy* mutants

Wild type and *vpy* mutants stained with nitroblue tetrazolium (NBT) to detect O_2^-. Strongest staining was observed in root tips (a-d) and lateral roots (e) of all genotypes (as indicated), independent of AM inoculation. In colonised areas of all genotypes (as indicated), strongest signal was detected in all fungal structures (f-j). White arrowheads indicate intercellular longitudinal hyphae; yellow arrowheads mark their tips, black arrowheads point to cell wall thickenings in hypodermal cells. m, root meristem; p, penetration peg. Size bars 100 µm in (a-e), 25 µm in (f-j).

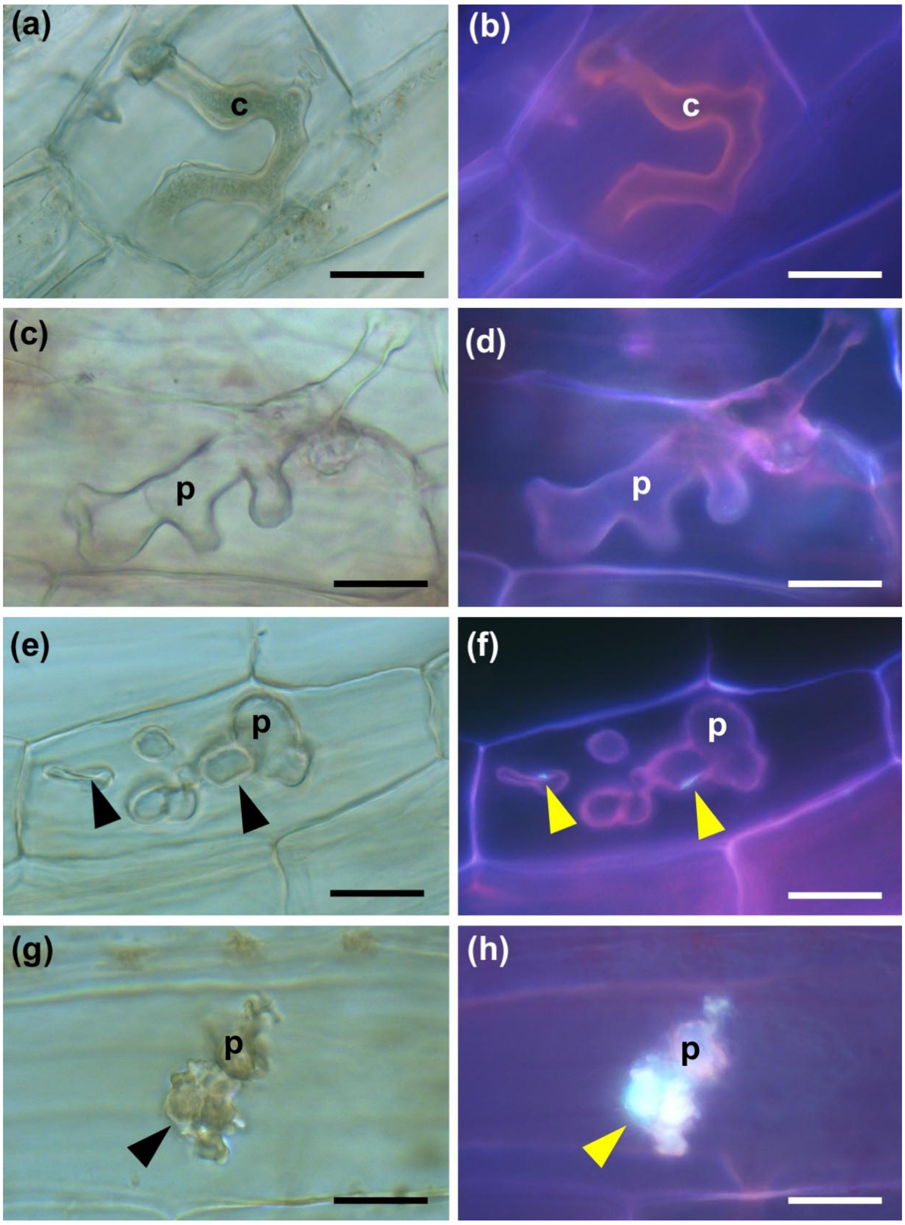

Figure S6. Callose accumulation associated with aborted fungal infection of *vpy-3*. Infection points of mycorrhizal wild type (a,b), and *vpy-3* (c-h), were evaluated after staining with aniline blue. (a,c,e,g) bright field images, (b,d,f,h) epifluorescence images of the same cells.

(a,b) Successful penetration of a wild type hypodermal cell. No callose can be observed around the hyphal coil.

(c,d) Penetrated cell of a *vpy-3* mutant root with a deformed hyphal peg but no callose deposition.

(e,f) Aborted fungal entry point into a hypodermal cell with weak callose accumulation (arrowheads).

(g,h) Aborted fungal entry into a hypodermal cell with the accumulation of callose-rich cell wall material (arrowhead). c, hyphal coil; p, penetration peg. Scale bars, 20 μm.

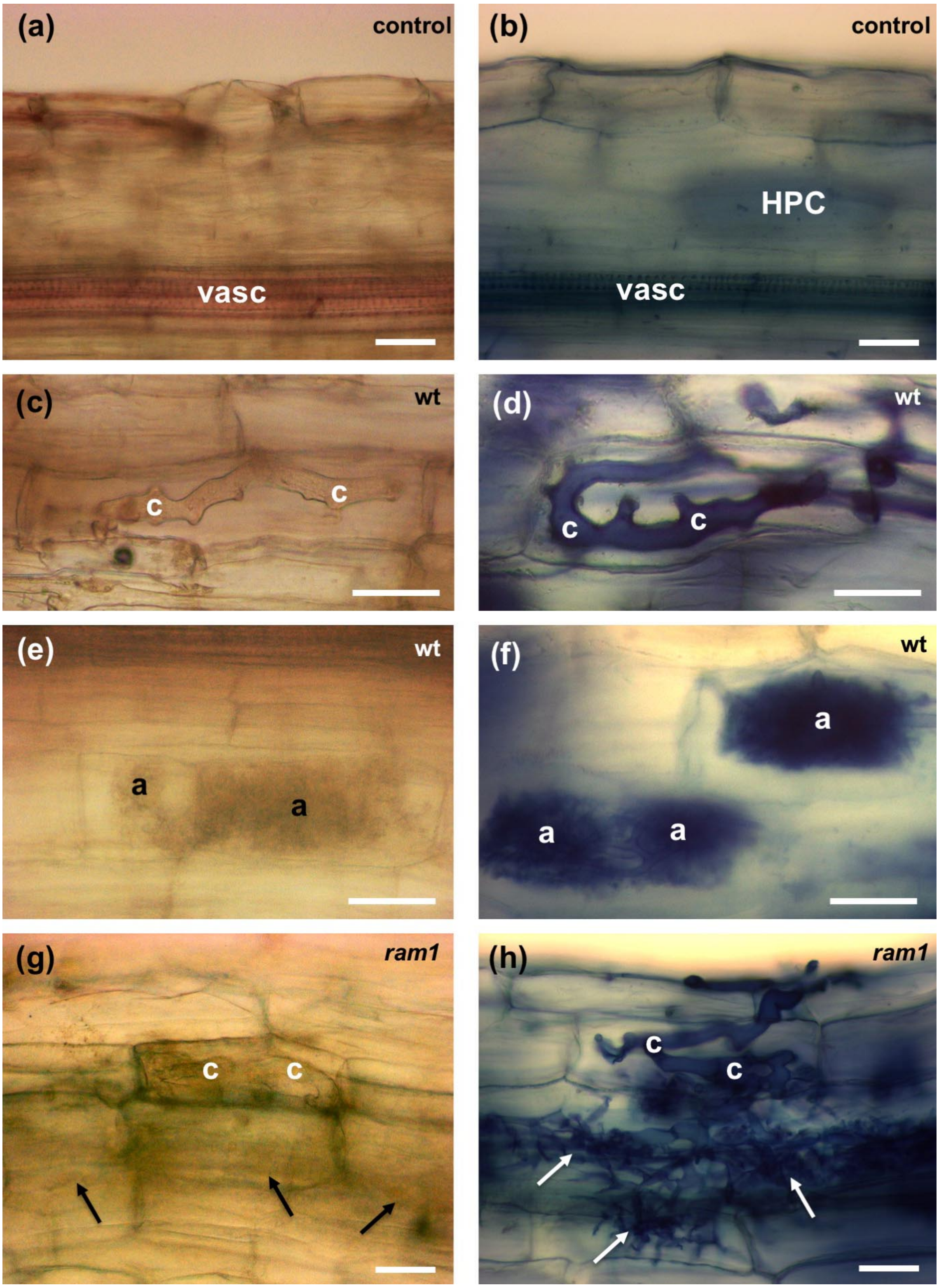

Figure S7. Lack of lignin accumulation in mycorrhizal *ram1* mutants
Non-colonised roots (a,b) or roots of colonised wild type (c-f), or colonised *ram1* mutants (g,h) were stained with phloroglucinol-HCl (a, c, e, g), or Trypan Blue (b, d, f, h), respectively. (a, b) non-colonised cells, (c,d) intracellular hypodermal coils, (e,f) arbuscules in the wild type, (g,h) infection unit with hyphal coils, and aberrant hyphal colonisation in the cortex (arrows). vasc, vascular bundles in the stele; HPC, hypodermal passage cell; c, hyphal coil; a, arbuscules. size bars 25 µm.

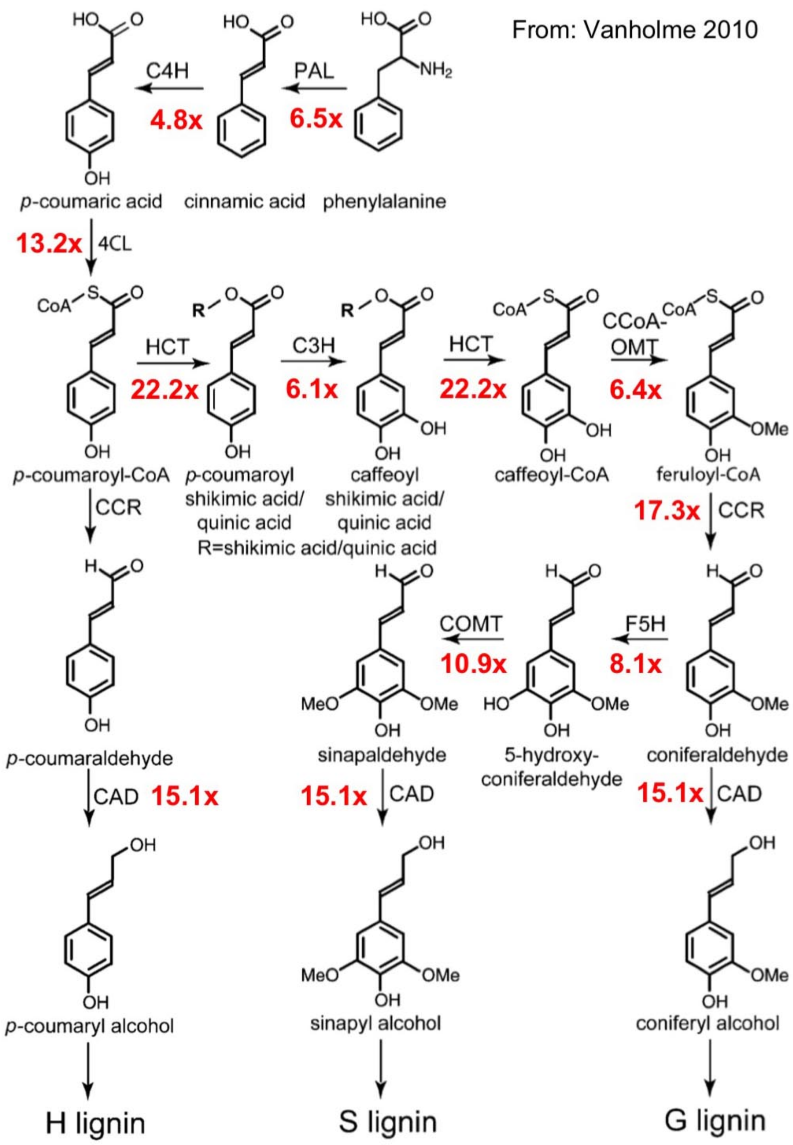

Figure S8. Concerted induction of lignin biosynthetic genes in mycorrhizal *vpy* mutants. The main biosynthetic route toward the monolignols p-coumaryl, coniferyl, and sinapyl alcohol is shown. Red numbers indicate the induction ratios of the respective gene functions calculated by averaging the induction ratios for the individual lignin-biosynthetic genes in a functional group (isozymes in Table 1), and by averaging the three *vpy* mutants. Pathway scheme after Vanholme *et al.* (2010).

PAL, phenylalanine ammonia-lyase; C4H, cinnamate 4-hydroxylase;
4CL, 4-coumarate:CoA ligase; C3H, p-coumarate 3-hydroxylase;
HCT, p-hydroxycinnamoyl-CoA:quinate/ shikimate p-hydroxycinnamoyltransferase;
CCoAOMT, caffeoyl-CoA O-methyltransferase; CCR, cinnamoyl-CoA reductase;
F5H, ferulate 5-hydroxylase; COMT, caffeic acid O-methyltransferase;
CAD, cinnamyl alcohol dehydrogenase.

Redrawn with permission from: Vanholme R, Demedts B, Morreel K, Ralph J, Boerjan W. 2010. Lignin biosynthesis and structure. *Plant Physiology* **153**(3): 895-905.

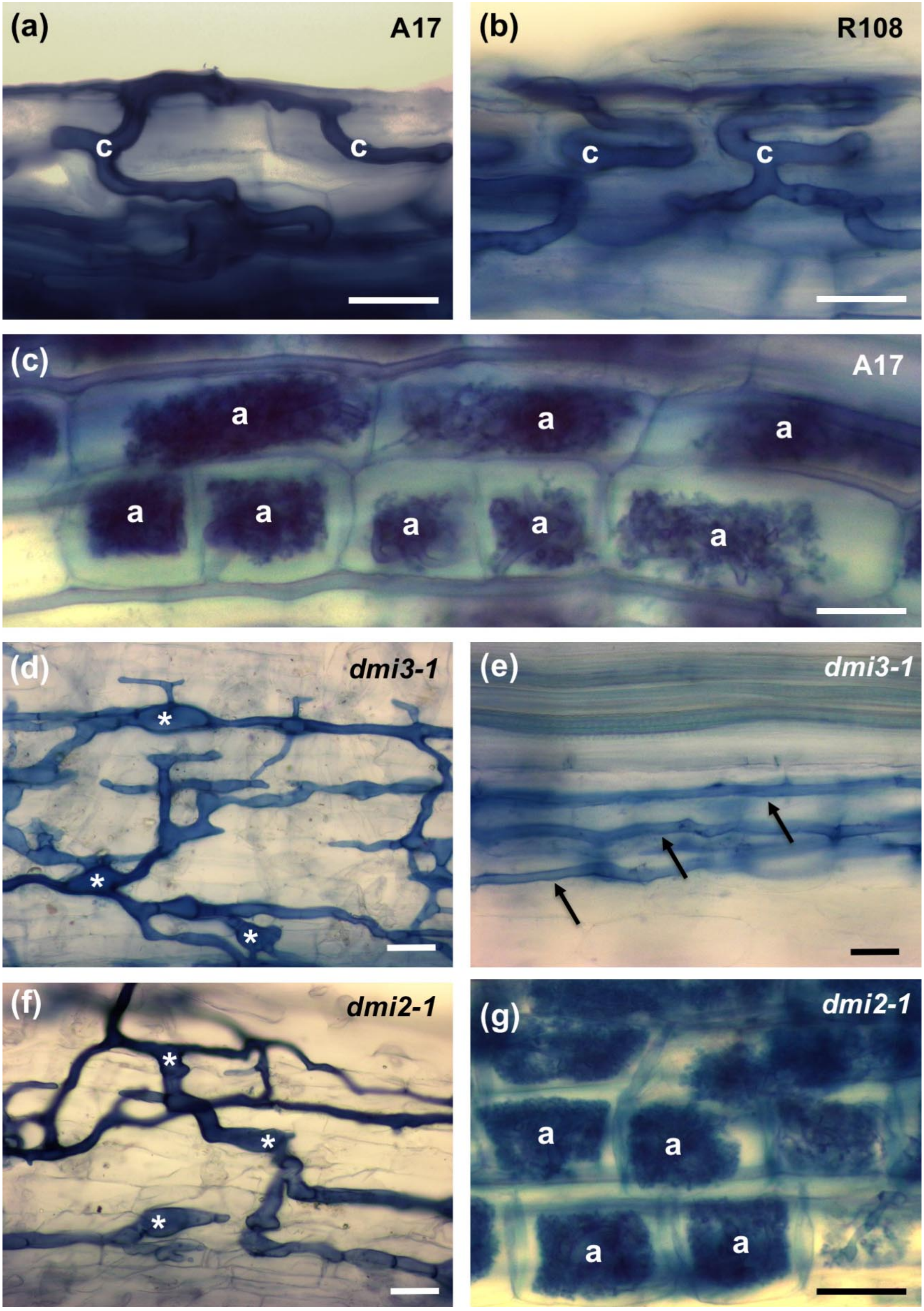

Figure S9. Mycorrhizal colonisation pattern in *M. truncatula sym* mutants
Mycorrhizal roots were stained with Trypan Blue. (a-c) wild type accessions A17 and R108 (as indicated). (d,e) *dmi3-1* mutant (defective in *CCaMK*), (f,g) *dmi2-1* mutant (defective in *SYMRK*). (a,b) Hyphal coils in hypodermal cells, (c) arbuscules in the cortex, (d,f) fungal hyphae growing on the root surface and attempted entry points with hyphopodia (asterisks), (e,g) cortical colonisation pattern with intercellular hyphae (arrows) (e), and intracellular arbuscules (g). a, arbuscules; c, hyphal coils. Size bars 25 µm.

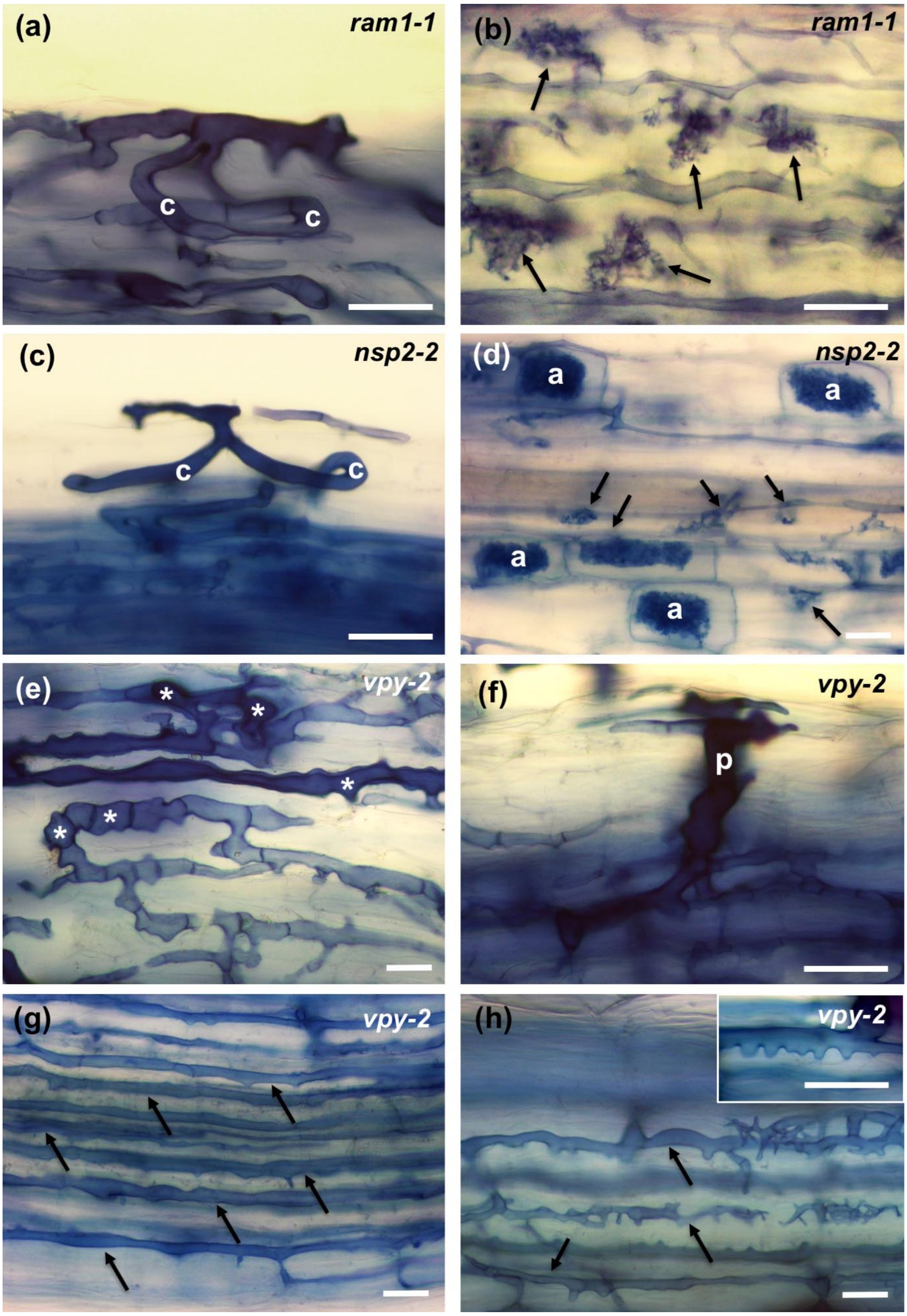

Figure S10. Mycorrhizal colonisation pattern in *M. truncatula sym* mutants

Mycorrhizal roots were stained with Trypan Blue. (a,b) *ram1* mutants; (c,d) *nsp2-2* mutants; (e-h), *vpy-2* mutants. (a,c) Entry point with hyphal coils; (b) Aberrant arbuscules (arrows) in the *ram1* mutant; (d) widely spaced normal arbuscules with intervening partially developed structures (arrows). (e) Hyphae growing on the root surface with hyphal swellings resembling hyphopodia (asterisks); (f) Fungal entry point with a thick penetration peg; (g,h) profusely growing intercellular hyphae (arrows), occasionally with lateral projections that may represent attempted cell invasion events (inset). a, arbuscules; c, hyphal coils; p, penetration peg. Size bars 25 µm.

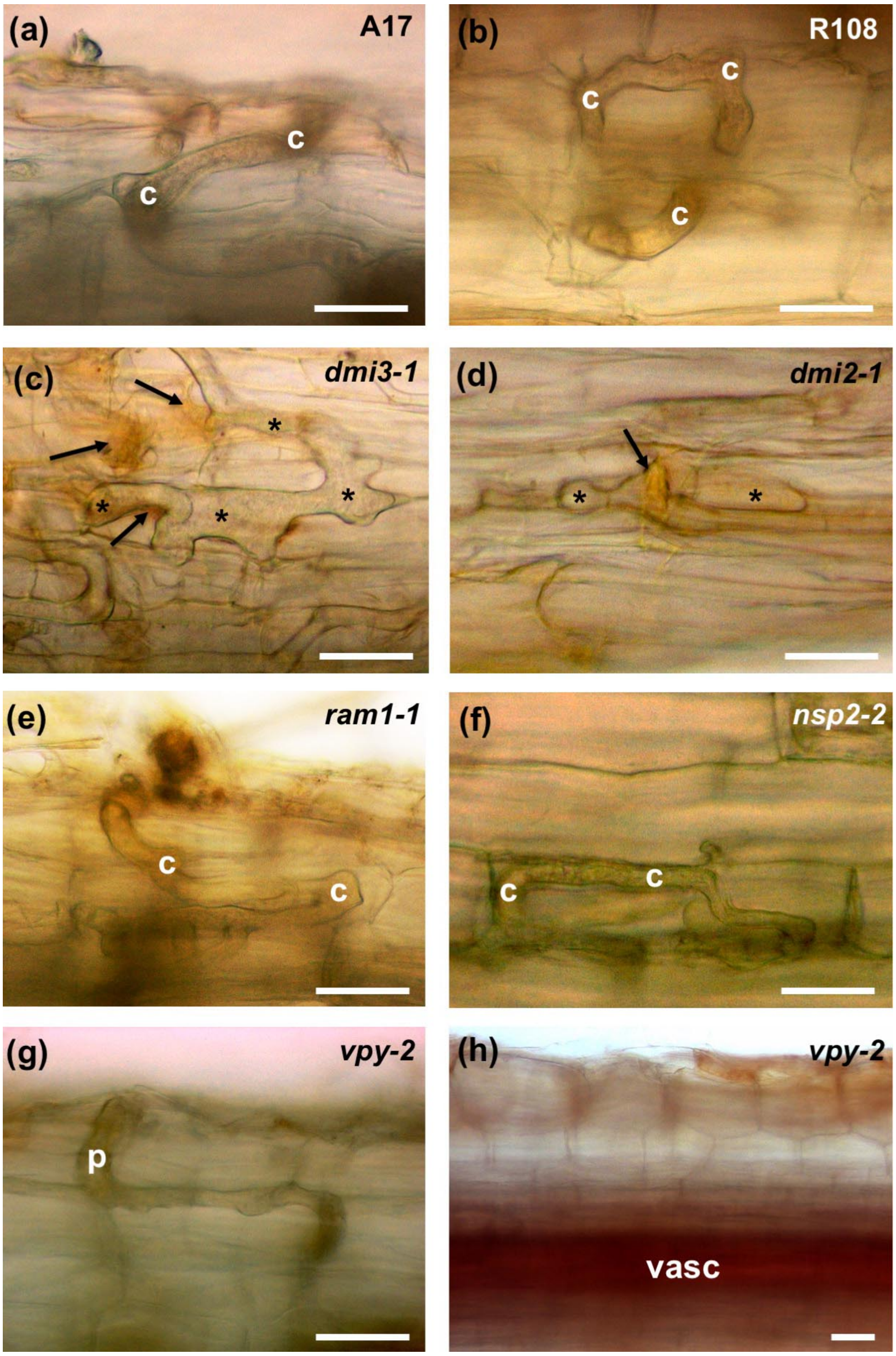

Figure S11. Lack of lignin accumulation at fungal entry points in *M. truncatula sym* mutants

Mycorrhizal roots were stained with phloroglucinol. (a,b) Wild type accessions A17 and R108, respectively. (c) *dmi3-1* mutant with attempted infection points represented by hyphal swellings (asterisks), and accumulation of brownish material in hosts cells (arrows); (d) *dmi2-1* with attempted infection points represented by hyphal swellings (asterisks), and accumulation of brownish material next to the fungal hyphae (arrow); (e) Hyphal coil in a hypodermal cell of a *ram1-1* mutant; (f) Hyphal coil in a hypodermal cell of an *nsp2-2* mutant; (g) Entry point in a *vpy-2* mutant by a hyphal penetration peg (p), and subsequent intercellular hypha; (h) Non-colonised root showing staining of the vasculature in the stele. c, hyphal coils; p, penetration peg; vasc, vasculature. Size bars 25 μm.

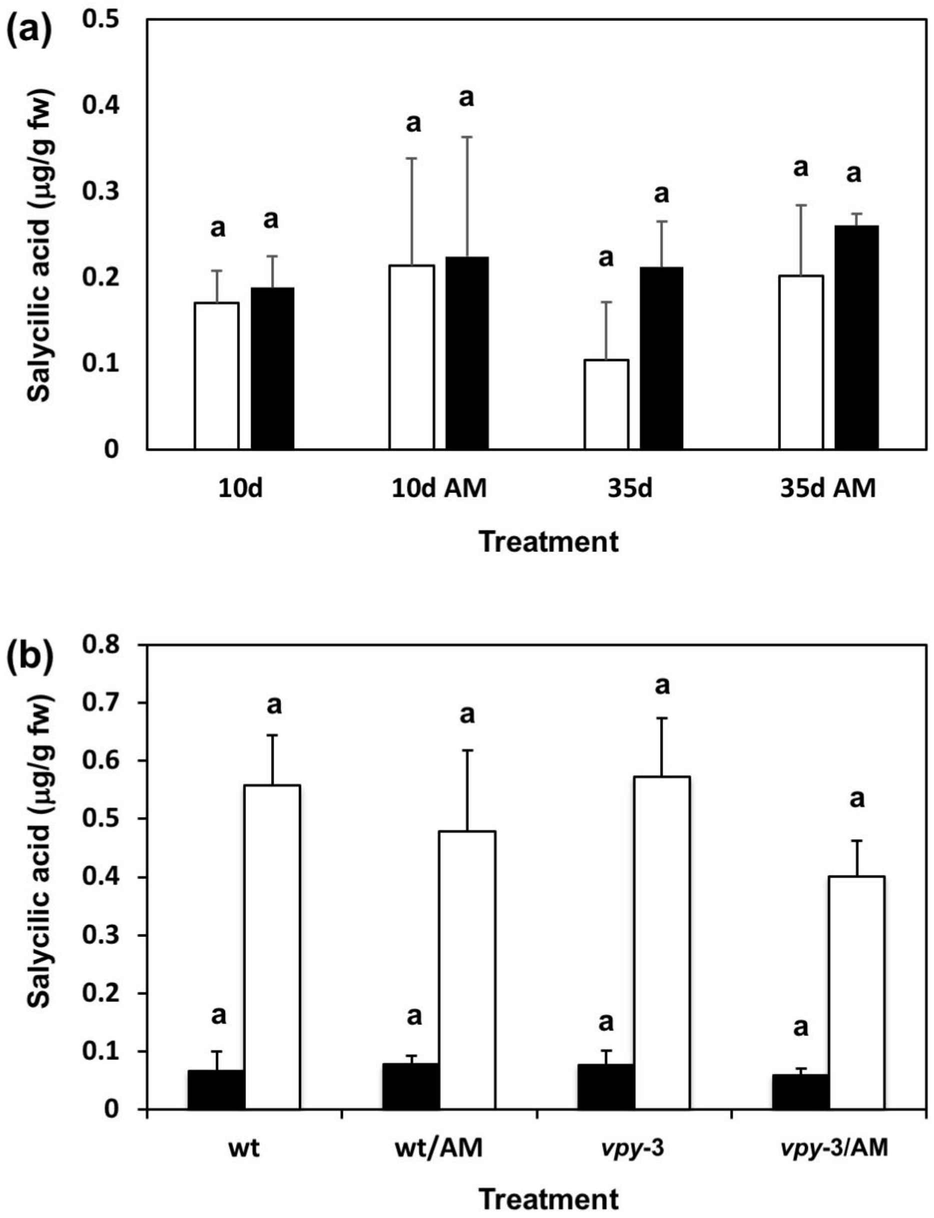

Figure S12. Salicylic acid levels in mycorrhizal wild type and *vpy-3* roots.

(a) Accumulation of free salicylic acid in wild type *P. hybrida* (white columns), and *vpy-3* plants (black columns) after 10 days (10d) and 35 days (35d) with or without inoculation with *R. irregularis* (AM).

(b) Free SA (black columns) and conjugated SA (white columns) in wild type and *vpy-3* roots with or without inoculation with *R. irregularis* (AM).

Columns represent means + Stdv (n=5). There was no significant difference between any of the treatments (one-way ANOVA).

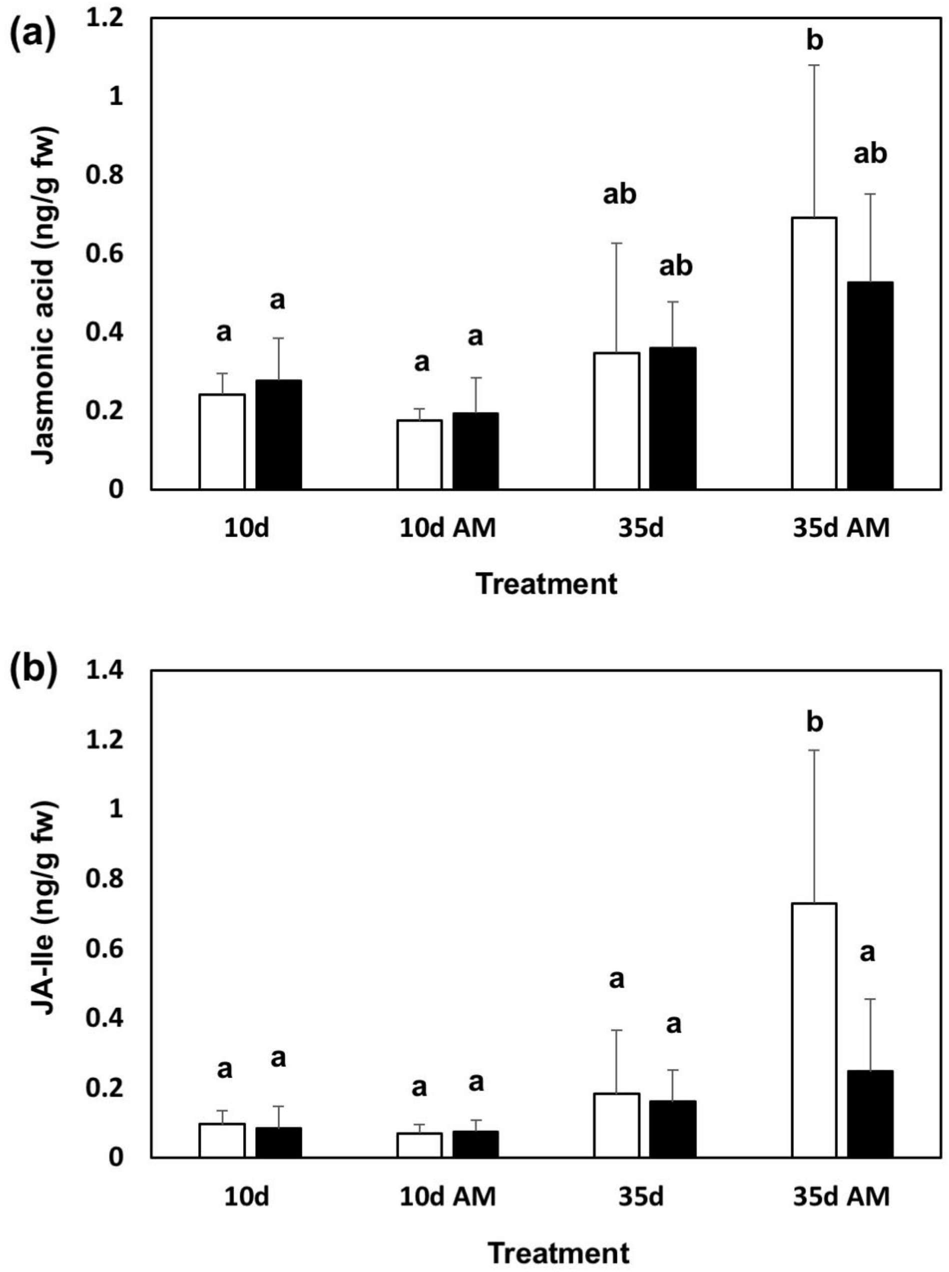

Figure S13. Jasmonic acid levels in mycorrhizal wild type and *vpy-3* roots.
(a) Accumulation of free jasmonic acid in wild type *P. hybrida* (white columns), and *vpy-3* mutants (black columns) after 10 days (10d) and 35 days (35d) with or without inoculation with *R. irregularis* (AM).
(b) Accumulation of isoleucine-conjugated jasmonic acid (JA-Ile) in wild type *P. hybrida* (white columns), and *vpy-3* mutants (black columns) after 10 days (10d) and 35 days (35d) with or without inoculation with *R. irregularis* (AM). Columns represent means + Stdv (n=5). Different letters indicate significant differences (one-way ANOVA).

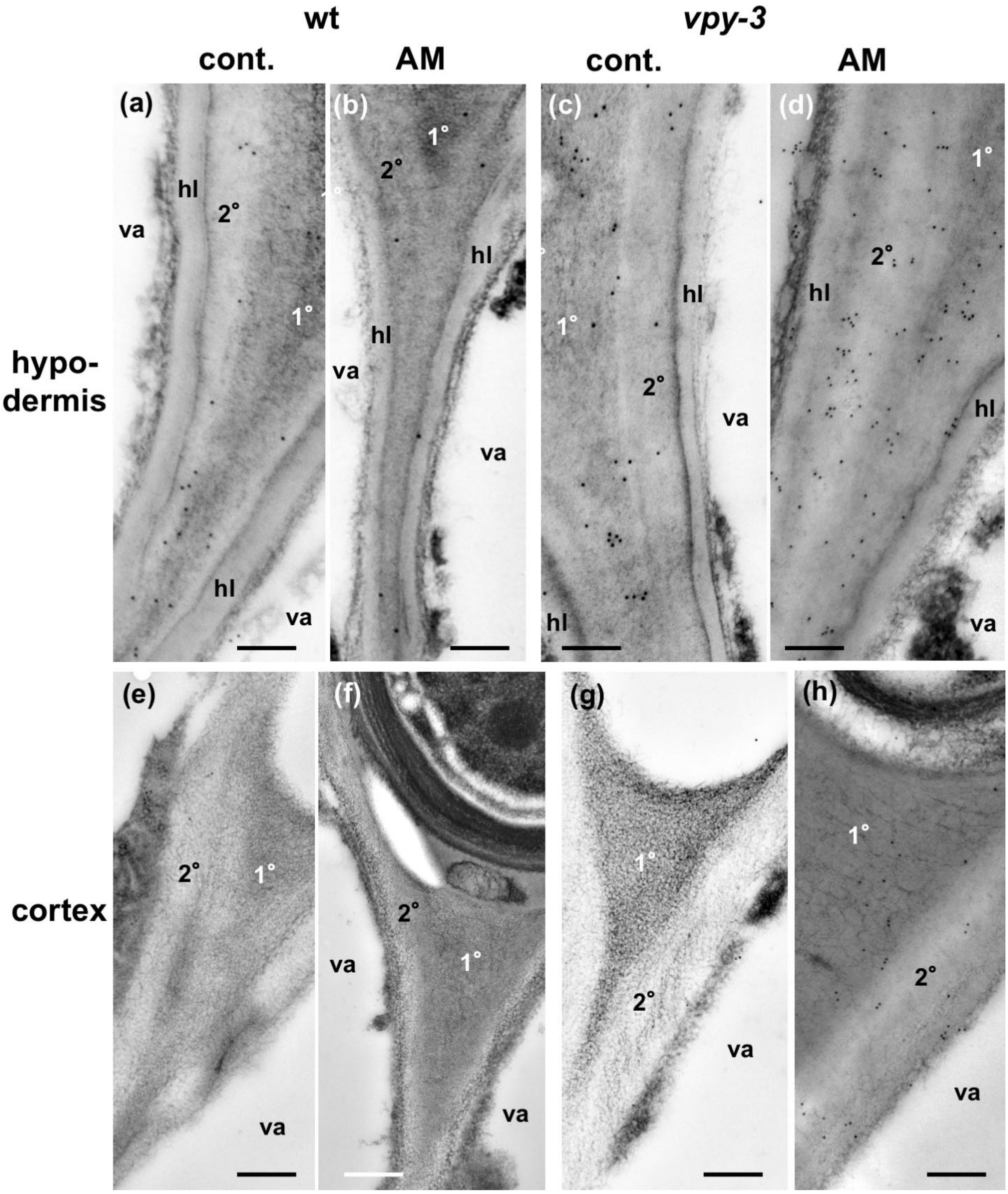

Figure S14. Immunocytochemical analysis of β-1,3-glucanase in mycorrhizal roots. Hypodermal cells (a-d), and cortical cells (e-h) of wild type (a,b,e,f), and *vpy-3* mutant roots (c,d,g,h). Non-inoculated control plants (a,c,e,g) and mycorrhizal plants (b,d,f,h) were analysed by immunogold staining with antibodies raised against tobacco β-1,3-glucanase. Low levels of immunogold were detected in hypodermal cells (a-d), while label was almost undetectable in cortex cells (e-h). va, plant vacuole; hl, hypodermal layer; 1°, primary cell wall; 2°, secondary cell wall. Scale bars, 250 nm.

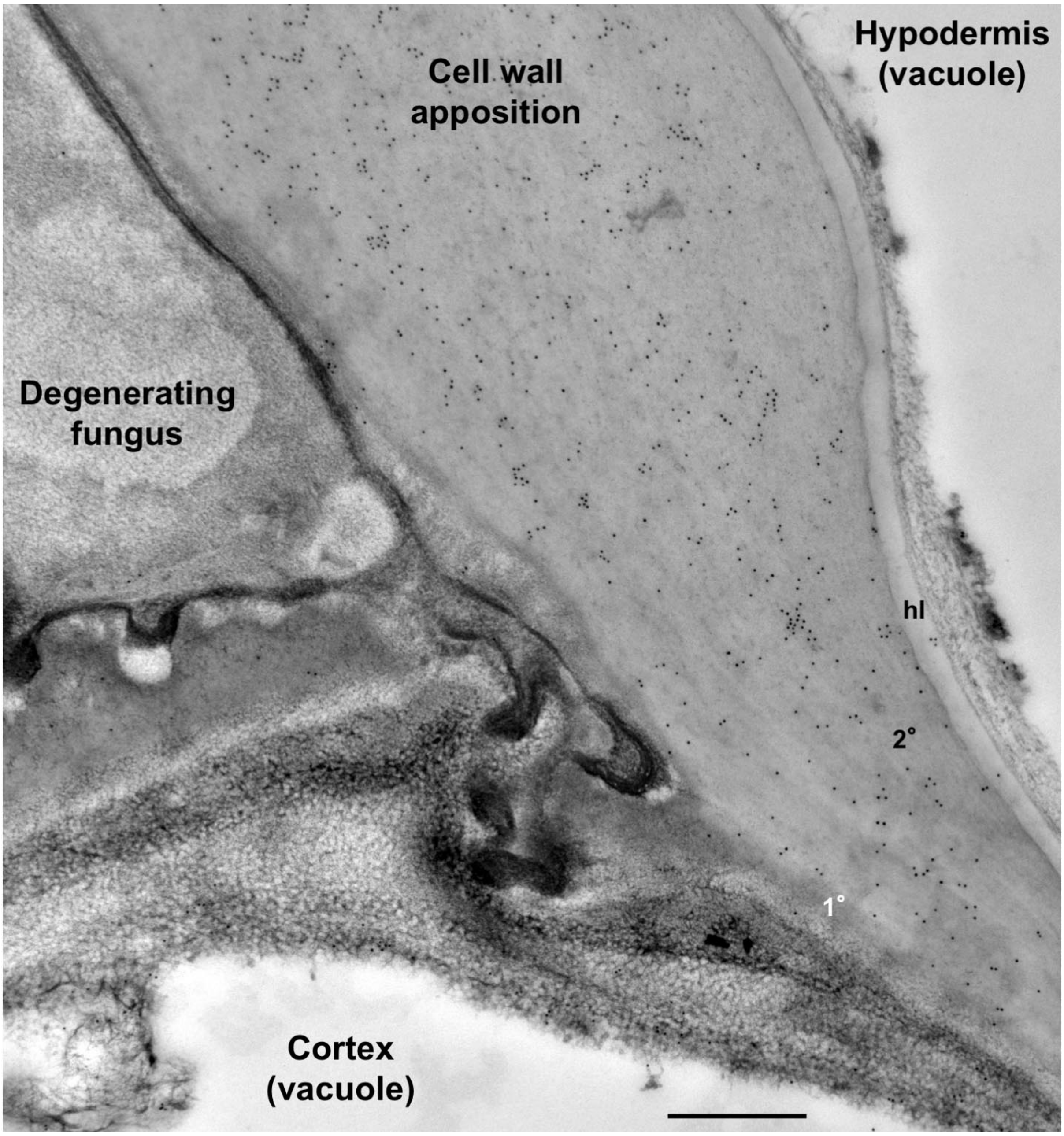

Figure S15. Accumulation of β-1,3-glucanase in a cell wall apposition of *vpy-3*.
Same sample as in Fig. 8d. An aborted fungal penetration hypha (degenerating fungus) grown next to a hypodermal cell (upper right) and a cortex cell (lower left). The hypodermal cell has formed a thick cell wall apposition that exhibits strong immunogold signal. hl, hypodermal layer; 1°, primary cell wall; 2°, secondary cell wall. Scale bar, 500 nm.

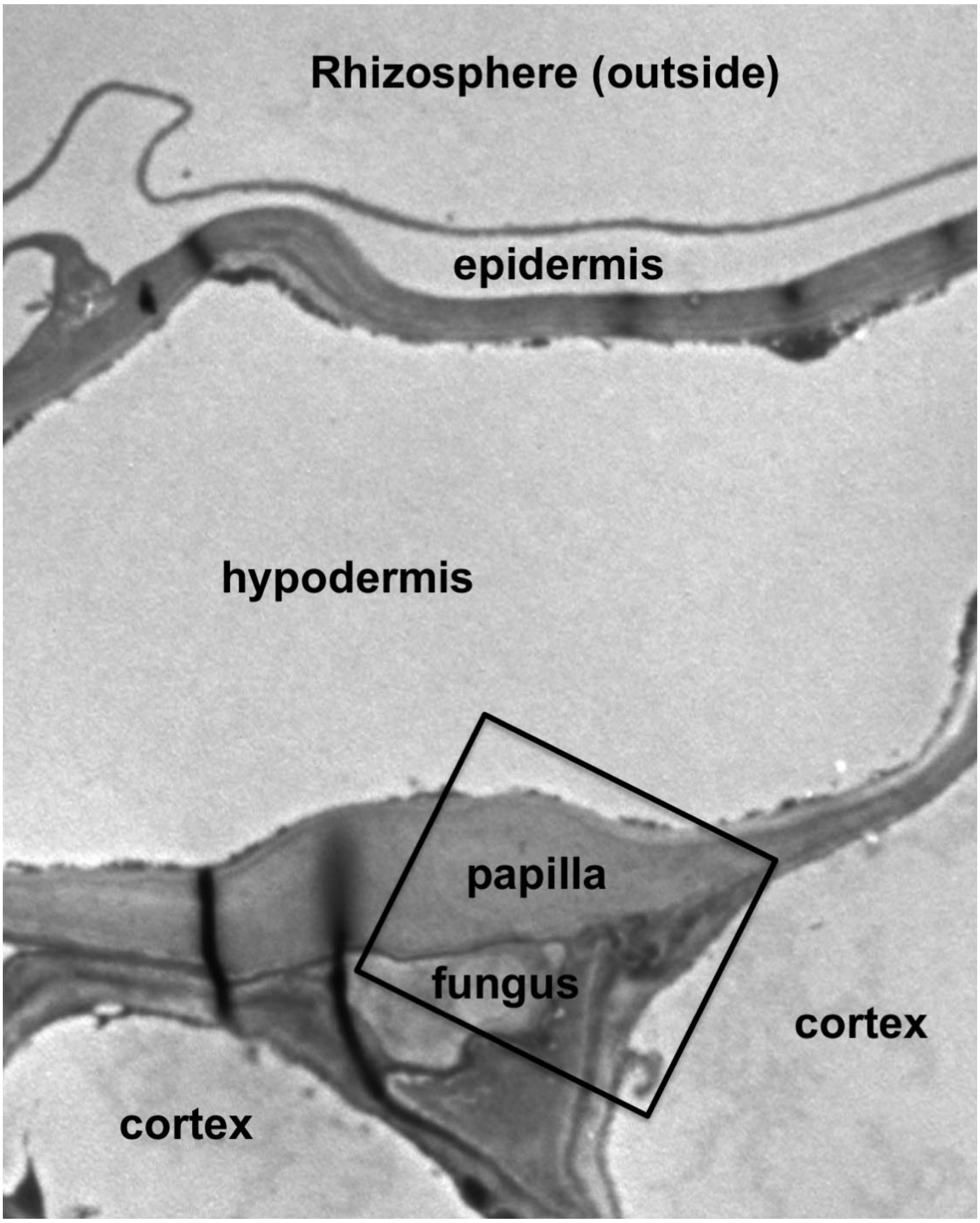

Figure S16. Low-magnification overview picture of the sample shown in Fig. S15.

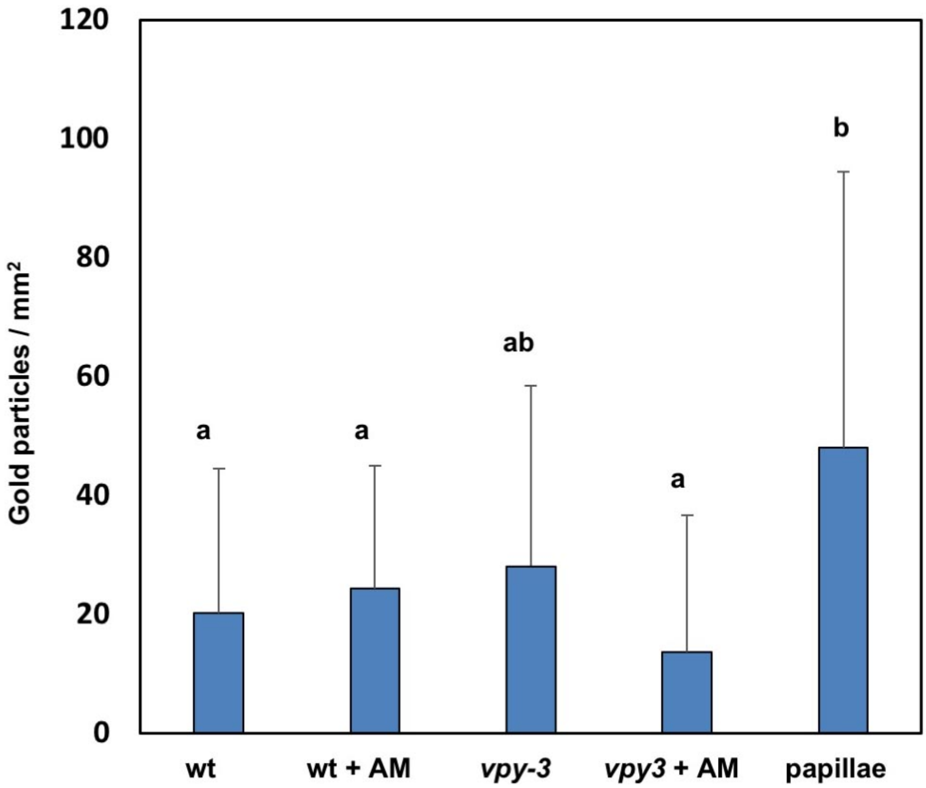

Figure S17. Quantification of β-1,3-glucanase immunogold signal
Immuno-gold signal was quantified in six representative TEM images as shown in Fig. S14 and Fig. S15 from two independent experiments. Gold particles were counted in the cell walls of hypodermal cells and expressed as relative density per unit of section area. Shown are the mean + Stdv. Different letters indicate significant differences (one-way ANOVA, n=6)

			Callose signal					
	Evaluated sites	infected cells	strong	weak	negative		callose all	% callose
wt	30	73	0	3	70		3	4.11
vpy-1	38	113	0	2	111		2	1.77
vpy-2	40	79	2	6	68		8	10.13
vpy-3	38	89	1	4	83		5	5.62

Table S1. Quantification of callose accumulation in *vpy* mutants
For each genotype at least 30 infection sites with at least one infected cell were chosen randomly. Presence of callose at infection sites was evaluated as shown in **Fig. S6** with strong or weak signal at penetration sites into hypodermal cells. The majority of infection sites did not exhibit any callose signal (negative). The column “callose all” refers to the accumulative cases in which either strong or weak signal was detected. The frequency of callose formation was not different in mutants compared to the wild type (Fisher’s Exact Test for count data).

	Gene ID	Forward Primer	Reverse Primer
PAL1	Peaxi162Scf00123g00096.1	CAATGGGTGCTAATGGTGAACTTCAT	CCTTCAATTCATCTTCGAAAGCTCCA
PAL2	Peaxi162Scf00488g00074.1	AAGATTGCAGCTTTTGAAGACGAGTT	ACCGTTCCATTCCTTGAGACATTCTA
PAL3	Peaxi162Scf00858g00215.1	CTAGTGGCCAAGAAAGTGTTGACAAT	AGCTCTCTCAATTTCCTTAGGCAGAA
4CL1	Peaxi162Scf00314g00086.1	TCCTAACATTTCTGATGCTGCTGTTG	GCAAGTCTAGCTCTCAAGTCCTTTCT
4CL2	Peaxi162Scf00207g00334.1	GATCCTGATACTGGGTGGCTACATAC	TTCTCCTGCTTGTTCATCTTTCATGG
4CL3	Peaxi162Scf00745g00865.1	GAGTCTCTTCTTGTAAGCCATCCAGA	CTGCTAGTTTGGCTCTCAGTTCTTTC
4CL4	Peaxi162Scf00610g00346.1	GAAAGATGAGGTTGCAGGAGAAGTTC	CAGACGGAGATTTTGGGATTGCTTC
CA4H	Peaxi162Scf00390g00225.1	GCCATTGATCACATTCTTGAAGCTCA	CCACCTTTCTCTGTAGTGTCGATCTT
C3H1	Peaxi162Scf00220g00211.1	TTGAAGGAGTGATGGATGAACAAGGA	AGGTCATATTTCTGTTGGAGGGTCAA
C3H2	Peaxi162Scf00975g00122.1	AGAACACACTCTTGCTAGGAAGGAA	GAGGCAATCTAGGAGTAGGAACAACT
COMT	Peaxi162Scf00912g00111.1	GATTGGAGTGATGAACATTGCCTGAA	GCTTCAAAGTCCTTCTCAGTCCTTTC
CCoAOMT1	Peaxi162Scf00450g00032.1	CCTGAACCCATGAAAGAGCTAAGAGA	GCTAGTATCTTGCCATCATCAGGAAG
CCoAOMT2	Peaxi162Scf00016g02023.1	TGCTTTGCCTGTTCTTGATCTAATGG	AATTTCAATTCTTGGATCAGCAGCCA
CCoAOMT3	Peaxi162Scf00316g00055.1	GAGGAGTGATAGGCTATGACAACACA	AAAATAAACTAGCTGAGACGCCTGC
HCT	Peaxi162Scf00045g02037.1	CACCAACCCCTTTTACGGATAACAAA	AGTTCCAAACAACAATCTCCCCATTC
CAD1	Peaxi162Scf00016g02329.1	GGAGTTATCAACACACCCTTGCAATT	CTTCCAGCAACATCGATCACAAATCT
CAD2	Peaxi162Scf00152g00245.1	TTTGGATTGACGCAGAGTGGATTAAG	ATGTAGTCAAATGAATCAGCAGCCTC
CCR1	Peaxi162Scf00332g00433.1	GTATGTGCATGTGAAGGATGTAGCTC	TCCAAACCCAAGTCCTTTAGCTTTTG
CCR2	Peaxi162Scf00207g00444.1	CAGTGCAGGCATATGTGGATGTTAAA	TGTTTCACTGGGGTAAATTCAAGACC
F5H1	Peaxi162Scf00083g01311.1	TATTAATTCATGGGCCATTGGACGTG	AAGTAAAACAATGAAGAAGGTGGGCC
F5H2	Peaxi162Scf00083g00156.1	TTATTAATTCATGGGCCATTGGACGC	AAGTAAAACAATGAAGAAGGTGGGCC
Actin7	Peaxi162Scf00025g02112.1	GAGGTTCCGTTGCCCAGA	CCCGCAGCTTCCATTCC
GAPDH	Peaxi162Scf00763g00325.1	GGAATCAACGGTTTTGGAAGAATTGGGCG	GGCCGTGGACACTGTCATACTTGAACA

Table S2. Primers used for qRT-PCR of lignin-related genes

Sequence identifiers and qRT-PCR primers for genes encoding predicted lignin biosynthetic enzymes. Gene IDs refer to gene names from the Solanaceae Genome Network database (https://solgenomics.net/) using the JBrowse function in the *Petunia axillaris* genome (https://solgenomics.net/organism/Petunia_axillaris/genome).

	AM(mut) / AM(wt)			c(mut) / c(wt)		
	*vpy1**	*vpy2*	*vpy3*	*vpy1**	*vpy2*	*vpy3*
PAL-1	**1.95**	**4.86**	**5.73**	1.27	1.29	0.94
PAL-2	**2.01**	**3.88**	**5.85**	1.28	1.67	1.52
PAL-3	**3.62**	**6.25**	**4.01**	**2.68**	**1.68**	1.28
4CL-1	**5.49**	**4.11**	**6.16**	0.82	1.60	1.14
4CL-2	**5.21**	**3.37**	**5.39**	0.81	1.52	1.19
4CL-3	**15.85**	**18.59**	**30.85**	0.35	1.05	0.79
4CL-4	**6.76**	**21.71**	**16.07**	1.07	**0.49**	0.98
CA4H	**4.36**	**5.67**	**3.87**	0.90	0.98	1.01
C3H-1	**5.00**	**5.63**	**6.24**	**0.51**	0.77	0.62
C3H-2	**3.41**	**3.16**	**5.10**	0.78	**1.90**	1.60
COMT	**3.26**	**7.00**	**8.19**	0.61	1.58	**1.39**
CCoAOMT-1	**6.82**	**4.39**	**4.24**	0.59	**1.88**	1.52
CCoAOMT-2	**3.06**	**4.89**	**7.20**	1.15	**1.80**	**1.53**
CCoAOMT-3	**3.21**	**17.65**	**37.97**	**3.73**	0.96	1.45
HCT	**2.42**	**5.84**	**12.15**	**2.70**	0.91	1.45
CAD1	**5.59**	**6.58**	**6.22**	1.47	0.96	1.08
CAD2	**8.20**	**15.16**	**26.65**	0.74	0.86	0.89
CCR1	**6.18**	**21.26**	**30.11**	0.83	0.91	1.03
CCR2	**6.18**	**6.64**	**5.80**	1.06	0.88	1.17
F5H2	**4.31**	**4.78**	**14.36**	1.01	1.33	1.18

Yellow: induction>2; orange: induction>4; red: induction>8; bold: p<0.05

Table S3. Induction of lignin-related genes in *vpy* mutants vs. wild type either in the mycorrhizal or non-mycorrhizal context.

Induction of lignin biosynthetic genes was determined by qRT-PCR with actin and glyceraldehyde-3-phosphate dehydrogenase (GAPDH) as reference genes. Values represent -fold induction ratios derived by dividing the expression values of mutants by the values from the wild type, either in mycorrhizal (left) or control conditions (right). All expression values represent the average of six biological replicates. Color shading represents induction >2-fold (yellow), >4-fold (orange), and >8-fold (red). Data represent two independent experiments, one with only *vpy-1* vs. wild type (asterisks), and one with *vpy-2* and *vpy-3* vs. wild type. Significant induction ratios are indicated by bold type font (one-way ANOVA, n=6).

	non-AM		AM	
	wt	*vpy-1*	wt	*vpy-1*
PAL-1	0.60	0.76	0.62	1.53
PAL-2	1.51	1.93	3.85	9.87
PAL-3	0.19	0.51	0.24	2.33
4CL-1	0.31	0.25	0.88	3.99
4CL-2	0.10	0.08	0.19	0.78
4CL-3	0.02	0.01	0.02	0.11
4CL-4	0.04	0.04	0.03	0.22
CA4H	0.55	0.50	0.53	2.09
C3H-1	0.11	0.05	0.17	0.42
C3H-2	0.00	0.00	0.00	0.01
COMT	0.19	0.12	0.21	0.42
CCoAOMT-1	0.34	0.20	0.35	1.41
CCoAOMT-2	0.51	0.59	0.60	2.12
CCoAOMT-3	0.00	0.00	0.00	0.01
HCT	0.00	0.00	0.00	0.01
CAD1	0.19	0.27	0.23	1.86
CAD2	0.06	0.04	0.06	0.38
CCR1	0.07	0.06	0.08	0.41
CCR2	0.16	0.17	0.13	0.84
F5H2	0.09	0.09	0.09	0.39

Table S4. Expression values for lignin-related genes for the experiment on *vpy-1* from the genes for which induction ratios were calculated in Table 1 and Table S3.

	non-AM			AM		
	wt	*vpy-2*	*vpy-3*	wt	*vpy-2*	*vpy-3*
PAL-1	0.37	0.48	0.35	0.44	2.74	2.37
PAL-2	1.52	2.53	2.30	3.14	20.24	27.86
PAL-3	0.20	0.34	0.26	0.27	2.83	1.38
4CL-1	0.37	0.59	0.42	0.69	4.49	4.81
4CL-2	0.10	0.14	0.11	0.13	0.68	0.86
4CL-3	0.02	0.02	0.01	0.02	0.33	0.41
4CL-4	0.03	0.01	0.03	0.02	0.21	0.32
CA4H	0.96	0.94	0.97	1.01	5.63	3.95
C3H-1	0.30	0.23	0.19	0.36	1.56	1.40
C3H-2	0.01	0.02	0.01	0.01	0.06	0.08
COMT	0.24	0.37	0.33	0.45	5.00	5.16
CCoAOMT-1	0.20	0.38	0.31	0.31	2.54	1.99
CCoAOMT-2	1.03	1.85	1.57	1.23	10.86	13.58
CCoAOMT-3	0.00	0.00	0.00	0.00	0.02	0.05
HCT	0.00	0.00	0.00	0.00	0.02	0.07
CAD1	0.09	0.09	0.10	0.21	1.33	1.42
CAD2	0.17	0.15	0.15	0.19	2.51	4.56
CCR1	0.03	0.03	0.03	0.04	0.86	1.37
CCR2	0.10	0.09	0.11	0.12	0.70	0.81
F5H2	0.03	0.04	0.04	0.03	0.20	0.53

Table S5. Expression values for lignin-related genes for the experiment on *vpy-2* and *vpy-3* from the genes for which induction ratios were calculated in Table 1 and Table S3.

Mutant	Affected gene	Genetic background	Reference
dmi2-1	*SYMRK*	A17	Endre et al., 2002
dmi3-1	*CCAMK*	A17	Lévy et al., 2004; Mitra et al., 2004
ram1-1	*RAM1*	A17	Gobbato et al., 2012
nsp2-2	*NSP*	A17	Kalo et al., 2005
vpy-2 (NF6898.86)	*VAPYRIN*	R108	Liu et al., 2019

Table S6. ***M. truncatula*** **mutants used in this study**

Endre G, Kereszt A, Kevei Z, Mihacea S, Kaló P, Kiss GB. 2002. A receptor kinase gene regulating symbiotic nodule development. *Nature* **417**(6892): 962-966.

Gobbato E, Marsh JF, Vernie T, Wang E, Maillet F, Kim J, Miller JB, Sun J, Bano SA, Ratet P, et al. 2012. A GRAS-type transcription factor with a specific function in mycorrhizal signaling. *Current Biology* **22**(23): 2236-2241.

Kalo P, Gleason C, Edwards A, Marsh J, Mitra RM, Hirsch S, Jakab J, Sims S, Long SR, Rogers J, et al. 2005. Nodulation signaling in legumes requires NSP2, a member of the GRAS family of transcriptional regulators. *Science* **308**(5729): 1786-1789.

Lévy J, Bres C, Geurts R, Chalhoub B, Kulikova O, Duc G, Journet EP, Ané JM, Lauber E, Bisseling T, et al. 2004. A putative Ca2+ and calmodulin-dependent protein kinase required for bacterial and fungal symbioses. *Science* **303**(5662): 1361-1364.

Liu CW, Breakspear A, Stacey N, Findlay K, Nakashima J, Ramakrishnan K, Liu MX, Xie F, Endre G, de Carvalho-Niebel F, et al. 2019. A protein complex required for polar growth of rhizobial infection threads. *Nature Communications* **10**: 2848.

Mitra RM, Gleason CA, Edwards A, Hadfield J, Downie JA, Oldroyd GED, Long SR. 2004. A Ca2+/calmodulin-dependent protein kinase required for symbiotic nodule development: Gene identification by transcript-based cloning. *Proceedings of The National Academy of Sciences of The United States of America* **101**(13): 4701-4705.

	Gene ID	Forward primer	Reverse primer
PR2a	Peaxi162Scf00032g10002.1	TTTGATGCCTTTTTGGATTCTATGTA	TCTCACTCTCGTCTCCTTTCTTATCA
PR2b	Peaxi162Scf00032g09002.1	CTGATGTCCCACTATCTTATGCACTT	TATTTTGCAGTTTGATTGGGATAGAA
PR2c	Peaxi162Scf00228g00020.1	AGTTATGGTAGGACTTCCCAATTCAG	GAGTTGCCAATCAAACTCATGTCTAC
PR3	Peaxi162Scf01261g00014.1	TATGACTTAGCTGGGAAAGCTATTGA	GTAATGACACCATAACCTGGTACACG
PR4b	Peaxi162Scf00016g31028.1	AAATACTAGGACAAGGGCTCAGGTAA	ATTGTCAACTACAGAAAGCAGAGGAA
PR4c	Peaxi162Scf00282g05024.1	CTGTGGTAGATGCTTGAGGAATAAGA	ATAGTTGACAATAAGGTGGCGTTGTT
PR4d	Peaxi162Scf00282g05022.1	TGAATGCTGTTAGTGCTTATTGTTCA	ATTGTCACCACAATTAACAAACTGGT
PR5a	Peaxi162Scf00714g01008.1	CACTTAGGGTACCTGGAGGATGTAAT	AGAGCCATAAGGACAGAAGACAACTT
PR6	Peaxi162Scf00714g02017.1	TCTTGCATCATTTACTCAACATCTCA	GTCACTGGAGAACCATTCAGTACAGT
PR7	Peaxi162Scf00620g03014.1	TCCACAGACATATACCAGAACTGTGA	CAAACACTACAGCAATTGGACTTCTT
PR9	Peaxi162Scf00192g00028.1	AAACACTACGTGGAATATGTCCTCAA	ATATTGCCCATTTTAATCATGGAGTT
PR14	Peaxi162Scf01238g02008.1	GGTTTAGCTGGTTGTCTTCCTTATTT	GTAAGGAATGTTAACACCACAAGCAG
PR17	Peaxi162Scf00016g24022.1	AGGAGAGAGATCACTGGCGTATTATT	GTTGCAGTAATCTAGAAATCGAGCAG
Actin7	Peaxi162Scf00025g02112.1	GAGGTTCCGTTGCCCAGA	CCCGCAGCTTCCATTCC
GAPDH	Peaxi162Scf00763g00325.1	GGAATCAACGGTTTTGGAAGAATTGGGCG	GGCCGTGGACACTGTCATACTTGAACA

Table S7. Primers used for qRT-PCR of PR genes

Sequence identifiers and qRT-PCR primers for genes encoding predicted PR proteins. Gene IDs refer to gene names from the Solanaceae Genome Network database (https://solgenomics.net/) using the JBrowse function in the *Petunia axillaris* genome (https://solgenomics.net/organism/Petunia_axillaris/genome).

	AM (mut)/AM(wt)			c(mut)/c(wt)		
	*vpy-1**	*vpy-2*	*vpy-3*	*vpy-1**	*vpy-2*	*vpy-3*
PR2a	**14.47**	**12.05**	**5.22**	0.87	0.66	2.27
PR2b	**4.18**	**12.30**	**17.38**	2.73	0.57	1.66
PR2c	1.26	0.87	0.70	0.95	1.16	0.61
PR3	**5.63**	**3.61**	**2.24**	0.53	1.31	1.65
PR4b	**4.72**	**7.66**	**2.39**	1.22	0.60	3.05
PR4c	**9.42**	**9.75**	**12.53**	0.92	1.04	1.33
PR4d	**0.43**	0.49	0.29	**9.78**	**2.14**	**4.23**
PR5a	0.17	0.29	0.33	**2.40**	0.98	**2.74**
PR6a	3.40	2.55	0.12	**6.71**	**15.97**	**15.59**
PR7	**6.29**	**6.45**	**1.35**	0.29	1.01	**1.48**
PR9	**3.52**	**1.22**	**4.97**	0.83	1.33	1.36
PR14	**5.90**	7.15	**6.56**	0.84	0.64	1.27
PR17	**6.67**	**6.18**	**1.76**	1.57	1.59	**2.25**

Yellow: induction>2; orange: induction>4; red: induction>8; bold: $p<0.05$

Table S8. Induction of pathogenesis-related (PR) genes in mutants vs. wild type either in the mycorrhizal or non-mycorrhizal context.
Induction of PR genes was determined by qRT-PCR with actin and glyceraldehyde-3-phosphate dehydrogenase (GAPDH) as reference genes. Values represent -fold induction ratios derived by dividing the expression values of mutants by the values from the wild type, either in mycorrhizal (left) or control conditions (right). All expression values represent the average of six biological replicates. Color shading represents induction >2-fold (yellow), >4-fold (orange), and >8-fold (red). Data represent two independent experiments, one with only *vpy-1* vs. wild type (asterisks), and one with *vpy-2* and *vpy-3* vs. wild type. Significant induction ratios are indicated by bold type font (one-way ANOVA, n=6).

	1h		4h	
	Chit	Pen	Chit	Pen
PR2a	1.38	2.93	0.61	0.82
PR2b	1.52	0.88	0.82	0.50
PR2c	1.38	1.35	**2.61**	**3.37**
PR3	1.91	1.33	0.93	**1.78**
PR4b	0.89	1.64	1.15	**2.44**
PR4c	2.04	0.88	0.97	0.92
PR4d	1.61	1.61	**5.45**	**5.02**
PR5a	1.45	2.21	1.54	**3.26**
PR6a	0.73	0.61	0.92	**2.84**
PR7	2.99	1.02	0.94	1.34
PR9	2.16	1.22	1.27	1.17
PR14	1.33	0.87	1.02	1.07
PR17	1.55	0.64	0.99	**1.96**

Yellow: induction>2; orange: induction>4; bold: $p<0.05$

Table S9. Induction of pathogenesis-related (PR) genes in wild type plants treated with fungal elicitor preparations.
Induction of PR genes was determined by qRT-PCR with actin and glyceraldehyde-3-phosphate dehydrogenase (GAPDH) as reference genes. Values represent -fold induction ratios derived by dividing the expression values of roots treated with chitin hydrolysate (Chit) or Penicillium preparation (Pen) for 1h or 4h by the values of control roots. All expression values represent the average of six biological replicates. Color shading represents induction >2-fold (yellow), and >4-fold (orange). Significant induction ratios are indicated by bold type font (one-way ANOVA, n=6).

	non-AM		AM	
	wt	*vpy-1*	*wt*	*vpy-1*
PR2a	0.0006	0.0005	0.0008	0.0098
PR2b	0.0002	0.0005	0.0003	0.0039
PR2c	0.2096	0.1985	0.3112	0.3707
PR3	0.0169	0.0090	0.0197	0.0593
PR4b	0.7377	0.8976	1.5068	8.6525
PR4c	0.0012	0.0011	0.0011	0.0094
PR4d	0.2050	2.0049	1.1415	4.7825
PR5a	2.0615	4.9515	13.7566	5.6603
PR6a	0.0222	0.1489	0.0399	0.9097
PR7	0.0099	0.0028	0.0421	0.0762
PR9	0.0013	0.0011	0.0092	0.0270
PR14	0.0024	0.0020	0.0060	0.0298
PR17	0.2138	0.3355	0.2889	3.0270

Table S10. Expression values for the experiment on *vpy-1* from the genes for which induction ratios were calculated in Table 2 and Table S8.

	non-AM			AM		
	wt	*vpy-2*	*vpy-3*	*wt*	*vpy-2*	*vpy-3*
PR2a	0.0008	0.0005	0.0018	0.0020	0.0158	0.0235
PR2b	0.0003	0.0001	0.0004	0.0004	0.0025	0.0103
PR2c	0.5561	0.6429	0.3387	0.7008	0.7030	0.2983
PR3	0.0119	0.0156	0.0196	0.0165	0.0782	0.0609
PR4b	0.3502	0.2093	1.0678	1.9080	8.7328	13.8989
PR4c	0.0032	0.0033	0.0042	0.0035	0.0355	0.0580
PR4d	0.1799	0.3843	0.7605	0.9921	1.0316	1.2303
PR5a	3.5041	3.4324	9.5838	12.1965	3.4931	10.8562
PR6a	0.0011	0.0183	0.0179	0.0152	0.6182	0.0281
PR7	0.0050	0.0050	0.0074	0.0260	0.1687	0.0522
PR9	0.0015	0.0020	0.0020	0.0079	0.0128	0.0532
PR14	0.0038	0.0024	0.0048	0.0114	0.0527	0.0949
PR17	0.1266	0.2012	0.2844	0.1771	1.7394	0.7017

Table S11. Expression values for the experiment on *vpy-2* and *vpy-3* from the genes for which induction ratios were calculated in Table 2 and Table S8.

	1h			4h		
	cont	CHIT	PEN	cont	CHIT	PEN
PR2a	0.004	0.006	0.013	0.016	0.010	0.013
PR2b	0.001	0.002	0.001	0.004	0.003	0.002
PR2c	1.825	2.514	2.467	6.542	17.080	22.041
PR3	0.006	0.011	0.007	0.009	0.008	0.015
PR4b	1.126	1.006	1.848	1.971	2.269	4.800
PR4c	0.002	0.004	0.002	0.007	0.006	0.006
PR4d	0.679	1.091	1.091	1.709	9.311	8.569
PR5a	5.513	7.994	12.161	19.895	30.722	64.844
PR6a	0.579	0.423	0.356	1.023	0.937	2.906
PR7	0.028	0.083	0.028	0.062	0.058	0.083
PR9	0.005	0.011	0.006	0.012	0.016	0.015
PR14	0.041	0.055	0.036	0.050	0.051	0.053
PR17	0.173	0.269	0.110	0.204	0.203	0.402

Table S12. Expression values from the experiment with wild type plants treated with elicitors from genes for which induction ratios were calculated in Table S9.

Table S13. p-values from statistical analyses and interaction report from two-way ANOVA tests

(see separate Excel sheet)
